# Describing the movement of molecules in reduced-dimension models

**DOI:** 10.1038/s42003-021-02200-3

**Published:** 2021-06-07

**Authors:** Natasha S. Savage

**Affiliations:** University of Liverpool, Liverpool, UK

**Keywords:** Computational biophysics, Computational models

## Abstract

When addressing spatial biological questions using mathematical models, symmetries within the system are often exploited to simplify the problem by reducing its physical dimension. In a reduced-dimension model molecular movement is restricted to the reduced dimension, changing the nature of molecular movement. This change in molecular movement can lead to quantitatively and even qualitatively different results in the full and reduced systems. Within this manuscript we discuss the condition under which restricted molecular movement in reduced-dimension models accurately approximates molecular movement in the full system. For those systems which do not satisfy the condition, we present a general method for approximating unrestricted molecular movement in reduced-dimension models. We will derive a mathematically robust, finite difference method for solving the 2D diffusion equation within a 1D reduced-dimension model. The methods described here can be used to improve the accuracy of many reduced-dimension models while retaining benefits of system simplification.

## Abstract (web summary)

Natasha Savage develops a general finite difference method for modeling 2D diffusion using a 1D reduced model. This method can be useful to improve the accuracy of many reduced-dimension models while retaining benefits of system simplification.

## Introduction

**Fig. 1:**
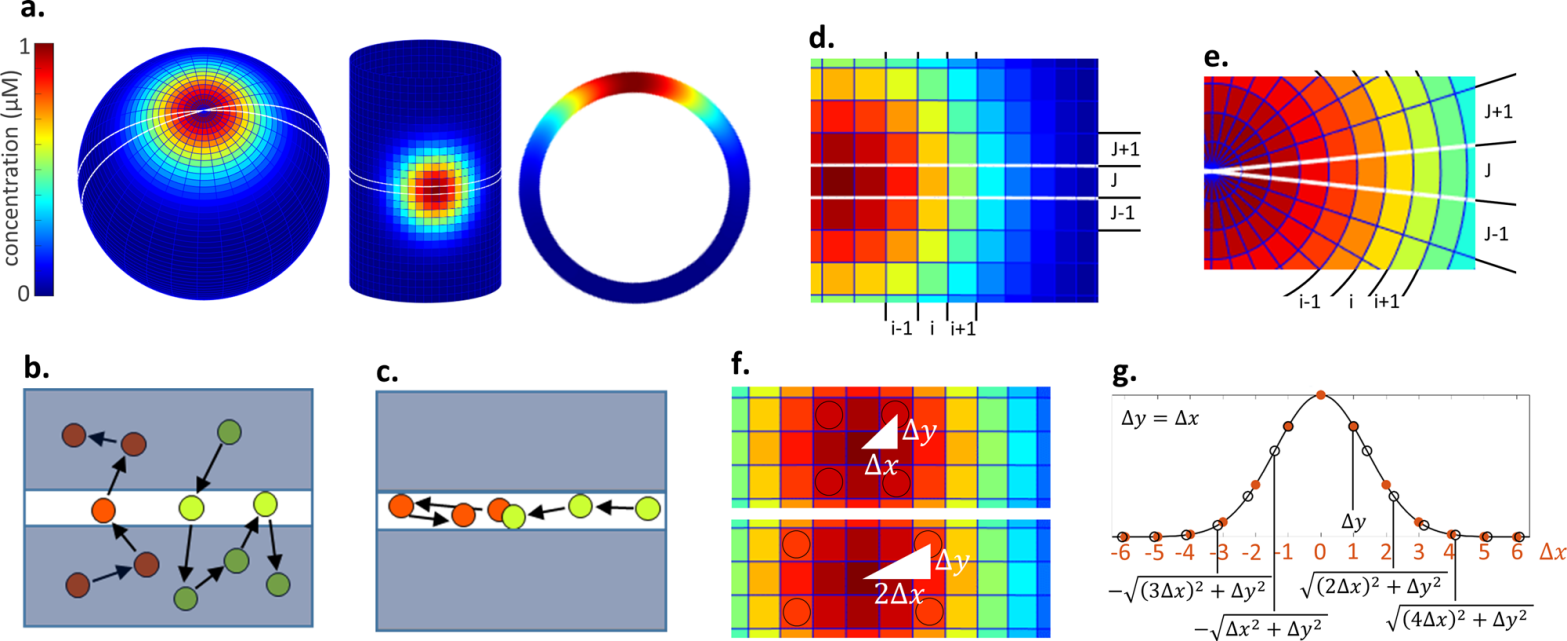
Dimension reduction. **a** Dimension reduction example showing a polarity patch on a spherical cell and body of an elongated cell with their reduced-dimension model representation, a stripe on a ring. White lines on 2D surfaces show the focal plane. **b** Molecules on a 2D surface moving through the focal plane. The focal plane is shown as a white stripe. **c** Molecules in a reduced-dimension model are restricted to the focal plane (white stripe). **d** Regular mesh over a polarity patch on the body of an elongated cell, the zero-flux assumption is not satisfied. **e** Spherical mesh over a polarity patch on a spherical cell. The zero-flux assumption is satisfied, ${u}_{i,J-1}^{\tau }={u}_{i,J}^{\tau }={u}_{i,J+1}^{\tau }$. **f** Regular mesh over a polarity patch on the body of an elongated cell. Distances from the centre of the patch of each point in rows $J\pm 1$ found using Pythagoras (white triangles and text). Concentrations in circles are the same because of radial symmetry. **g** Using interpolation (black line) on the concentrations in the focal plane (orange dots) to estimate ${\widetilde{u}}_{i,J\pm 1}^{\tau }$ (black open circles).

Simple models of complex systems are an invaluable tool for gaining conceptual insight into biological mechanism^1,2^. In spatial models a powerful simplification technique often used is to exploit symmetries within a biological system’s geometry and patterning to reduce the physical dimension of the problem. For example, consider a single cell and the formation of a concentrated patch of membrane-associated proteins, a polarity patch, this system has radial symmetry and so mechanisms controlling patch formation could be explored by considering a one-dimensional (1D) slice through the centre of the patch, rather than considering the entire two-dimensional (2D) membrane^3–8^ (Fig. 1a). When addressing questions which include cytoplasmic gradients, for example, a system’s dimension is often reduced from 3D to 2D^1,9–13^, or even 1D^1,2,14–16^. An analogy for reduced-dimension models is the focal plane in microscopy: Analysis is performed on data acquired from a representative slice through the system, then results are inferred onto the entire cell or tissue. All spatial models contain a description of molecular movement. Molecular movement in reduced-dimension models is restricted to the focal plane (compare Fig. 1b and c). Thus, when one reduces the dimension of a system and restricts molecular movement to the reduced dimension, they are changing the geometry of the problem. It is understood that cell geometry influences molecular movement and patterning^11,13,17–19^. Here we present a general methodology that enables a reduced-dimension model to take the system’s full geometry into account, by using it to estimate the movement of molecules through the focal plane. We go on to use this general methodology to derive a simple numerical method (the 1D-uFDM) that can be used to solve the 2D diffusion equation in a 1D reduced-dimension model.

## Results

Within this manuscript, we will consider the full system to be a 2D surface, a membrane, and the reduced-dimension model to be a 1D ring (Fig. 1a). Molecular movement will be diffusive. Throughout the manuscript 2D and 1D solutions are compared to analyse and illustrate the accuracy of the reduced-dimension method being proposed. A user of this method would not generate the full dimension model, they would only generate the simplified reduced-dimension model.

### Reduced-dimension models have a zero-flux assumption

In order to use a numerical method to calculate changes in molecular concentrations over space and time, the space and time must be discretised. For a 2D space, discretisation is achieved by drawing a mesh over the space (Fig. 1a, d, e). The concentration within each discretised space is represented by a single value and this value is used in the numerical method. Discretisation of time is analogous to collecting time course data, one defines a time step, $\triangle t$ seconds, and collects data at a number of time points $\triangle t$ seconds apart. The finite difference method is a commonly used method for calculating molecular concentrations over discretised space and time^20,21^.

To investigate any inbuilt assumptions of reduced-dimension models we compared the finite difference solution to the diffusion equation in 2D and 1D. For the 2D solution cover the 2D membrane with a regular mesh (Fig. 1d). The mesh points along the $x$-axis are labelled $i={1,2},\ldots ,N$ and are distance $\triangle x\,\upmu {\mathrm{m}}$ apart, the mesh points along the $y$-axis are labelled $j={\mathrm{1,2}},\ldots ,M$ and are distance $\triangle y\,\upmu {\mathrm{m}}$ apart. Let ${u}_{i,j}^{\tau }$ represent the concentration of molecule $u$ on the membrane at point $\left(i,j\right)$ and time point $\tau$, $\tau ={\mathrm{0,1}},2,3,\ldots ,$ time points are $\triangle t$ seconds apart. The change in concentration of ${u}_{i,j}^{\tau }$ over time, as a result of diffusive movement, is described by the solution to the 2D diffusion equation. The explicit 2D finite difference scheme (2D-FDM) used to solve the 2D diffusion equation on a regular mesh, with diffusion coefficient $D\,{\upmu {\mathrm{m}}}^{2}{\mathrm{s}}^{-1}$, is^20,21^

Explicit 2D-FDM:

1 $${u}_{i,j}^{\tau +1}={u}_{i,j}^{\tau }+\frac{\triangle t}{{\triangle x}^{2}}D\left({u}_{i-1,j}^{\tau }-2{u}_{i,j}^{\tau }+{u}_{i+1,j}^{\tau }\right)+\frac{\triangle t}{{\triangle y}^{2}}D\left({u}_{i,j-1}^{\tau }-2{u}_{i,j}^{\tau }+{u}_{i,j+1}^{\tau }\right)$$

The finite difference method is an iterative method. Thus, one must know the concentrations of molecule $u$ at all points of the mesh at time point $\tau =0$. We denote these initial concentrations as ${u}_{i,j}^{0}$. The initial concentrations (${u}_{i,j}^{0}$) are substituted into Eq. (1) to calculate ${u}_{i,j}^{1}$. ${u}_{i,j}^{1}$ are the concentrations at time point $1$. The time at time point $1$ is $\Delta t$ seconds. The concentrations ${u}_{i,j}^{1}$ are then substituted into Eq. (1) to calculate ${u}_{i,j}^{2}$, the concentrations at time point $2$, $2\Delta t$ seconds. This iterative process continues until adequate time course data are calculated. The calculated time course data are referred to as the solution to the finite difference method.

Note that in order to calculate the concentrations ${u}_{i,j}^{\tau +1}$ the 2D-FDM calculates molecular movement along the $x$-axis and $y$-axis separately. Molecular movement along the $x$-axis is calculated by the term $\frac{\triangle t}{{\triangle x}^{2}}D\big({u}_{i-1,j}^{\tau }-2{u}_{i,j}^{\tau }+{u}_{i+1,j}^{\tau }\big)$, molecular movement along the $y$-axis is calculated by the term $\frac{\triangle t}{{\triangle y}^{2}}D\big({u}_{i,j-1}^{\tau }-2{u}_{i,j}^{\tau }+{u}_{i,j+1}^{\tau }\big)$.

To build a 1D reduced-dimension model describing the diffusive movement of $u$ on a 2D surface, the focal plane is set to run through the axis of symmetry of $u$ and the membrane (Fig. 1a). Molecule movement through the focal plane is then approximated by the solution to the 1D diffusion equation. Recall the 2D mesh, assume that the focal plane is set along the $x$-axis at row $j=J$ (Fig. 1d). The explicit 1D finite difference scheme (1D-FDM) used to solve the 1D diffusion equation is^20,21^

Explicit 1D-FDM:

2 $${u}_{i,J}^{\tau +1}={u}_{i,J}^{\tau }+\tfrac{\varDelta t}{\varDelta {x}^{2}}D\Big({u}_{i-1,J}^{\tau }-2{u}_{i,J}^{\tau }+{u}_{i+1,J}^{\tau }\Big)$$

As the 1D reduced-dimension model only contains information about concentrations on row $J$ the 1D-FDM contains no terms for calculating the movement of $u$ along the $y$-axis (compare the 2D and 1D-FDMs, Eqs. 1 and 2). Thus, molecular movement in the 1D reduced-dimension model is modelled as though it is restricted to the focal plane (Fig. 1c). An inbuilt assumption of the 1D reduced-dimension model is that $\big({u}_{i,J-1}^{\tau }-2{u}_{i,J}^{\tau }+{u}_{i,J+1}^{\tau }\big)=0$, the number of molecules leaving the focal plane, $-2{u}_{i,J}^{\tau }$, is equal to the number of molecules entering it, ${u}_{i,J-1}^{\tau }+{u}_{i,J+1}^{\tau }$, at all points in space, $i$, for all time, $\tau$: There is zero-flux through the focal plane.

### Conditions under which the zero-flux assumption is valid

The zero-flux assumption is valid for a subset of reduced-dimension models, those for which a mesh can be drawn such that the zero-flux assumption holds. For example, consider the formation of a polarity patch on a spherical cell, one could construct a spherical mesh with a pole located at the centre of the patch (Fig. 1a). Because of the placing of the mesh concentrations ${u}_{i,J-1}^{\tau }$, ${u}_{i,J}^{\tau }$ and ${u}_{i,J+1}^{\tau }$ are equal and the zero-flux assumption holds (Fig. 1e). Thus, molecular movement in a 1D reduced-dimension model of this system would be accurately described by the 1D diffusion equation.

An example of a system for which the zero-flux assumption does not hold is the formation of a polarity patch along the body of an elongated cell (Fig. 1a). To solve the diffusion equation here a square mesh is constructed on the cell surface. As a polarity patch is radial and the mesh is square a focal plane cannot be found such that a 1D reduced-dimension model would obey the zero-flux assumption (Fig. 1d). Thus, the 1D diffusion equation would give an inaccurate approximation of 2D molecular movement through the focal plane.

For the modeller, the validity of the zero-flux assumption can be ascertained without generating a solution for the full system and calculating molecular movement through the focal plane. The modeller can consider the symmetries in the full system and the form of the full mesh using cartoons, as in Fig. 1. From the cartoons one can estimate whether or not the zero-flux assumption holds. This cartoon estimation of zero-flux is not dissimilar to the estimation made by the modeller when deciding whether or not it is appropriate to reduce a system’s dimension.

### A general method for calculating molecular movement into and out of the focal plane

Here we present a general methodology which can be used to increase the accuracy of reduced-dimension models that do not satisfy the zero-flux assumption: If it is possible to estimate the concentrations either side of the focal plane (for example, terms ${u}_{i,J+1}^{\tau }$ and ${u}_{i,J-1}^{\tau }$ in the explicit 2D-FDM equation) using the concentrations on the focal plane (${u}_{i,J}^{\tau }$ in our example), then we can estimate molecular movement into and out of the focal plane. In order to reduce a system’s dimension, the system must exhibit symmetry. Therefore, by definition, the concentration profile in the focal plane (i.e. in the dimensionally reduced model) represents the concentration profile of the full system and thus holds information about the concentrations at every point in the full system. As such, the information in the focal plane can be used to calculate concentrations either side of the focal plane in any system for which dimension reduction is possible.

Below we provide a proof of principle for the general method. We derive a mathematically robust numerical method which can be used to improve the accuracy of a subset of reduced-dimension models. Namely, diffusive movement in 1D reduced-dimension models that do not satisfy the zero-flux assumption and exhibit radial molecular dynamics. This proof of principle does not represent the limits of the general method for estimating molecular movement into and out of the focal plane. As discussed above, the general method can be used for systems of any dimension and concentration profiles that do not exhibit radial symmetry. Furthermore, molecular movement need not be governed by diffusion.

### Solving the 2D diffusion equation in a 1D reduced-dimension model

We will use the general methodology to construct a FDM that solves the 2D diffusion equation in a 1D reduced-dimension model. Consider again the polarity patch on the body of an elongated cell (Fig. 1a, d, f). The 2D polarity patch has radial symmetry. A property of radial symmetry is that the concentration profile along all lines running through the centre of the patch is identical. When we reduce the dimension of this system to 1D, we position the focal plane such that the 1D model calculates the concentration profile along one line running through the centre of the 2D patch (row $J$). As the concentration profile on all lines running through the centre of the patch is identical, we can use the concentration profile in the 1D model to calculate the concentrations at all points on the 2D surface. The explicit 2D-FDM (Eq. 1) contains two concentrations that are not on the focal plane, ${u}_{i,J+1}^{\tau }$ and ${u}_{i,J-1}^{\tau }$. Thus, to calculate molecular movement into and out of the focal plane, row $J$, and solve the 2D diffusion equation in a 1D reduced-dimension model, we need only estimate concentrations on either side of the focal plane, rows $J\pm 1$.

Let ${\tilde{u}}_{i,J\pm 1}^{\tau }$ denote the estimated concentrations on rows $J\pm 1$. ${\tilde{u}}_{i,J\pm 1}^{\tau }$ can be estimated from the concentrations of molecule $u$ along the focal plane, ${u}_{i,J}^{\tau }$, using interpolation. The interpolation mesh points are found using Pythagoras’ theorem (Fig. 1f, g, “Methods: Generating the interpolation mesh in 1D”). In order to use Pythagoras to calculate the interpolation mesh points, the modeller has to set a value for $\Delta y$. In the illustrative examples below, we have set $\Delta y=\Delta x$. Analysis on the accuracy of estimating concentrations either side of the focal plane and the choice of $\Delta x$, $\Delta y$, can be found in Supplementary: Accuracy of estimating concentrations at phantom points using interpolation. Further discussion on the derivation of the explicit 1D finite difference diffusion equation with unrestricted movement (explicit 1D-uFDM) can be found in “Methods: Explicit 1D-uFDM derivation”. The 1D-uFDM is

Explicit 1D-uFDM:

3 $${u}_{i}^{\tau +1}={u}_{i}^{\tau }+\tfrac{\varDelta t}{\varDelta {x}^{2}}D\Big({u}_{i-1}^{\tau }-2{u}_{i+1}^{\tau }+{u}_{i+1}^{\tau }\Big)+\tfrac{\varDelta t}{\varDelta {x}^{2}}D\Big({\tilde{u}}_{i,J-1}^{\tau }-2{u}_{i}^{\tau }+{\tilde{u}}_{i,J+1}^{\tau }\Big)$$

See “Methods: Explicit 1D-uFDM solution” for the solution to the explicit 1D-uFDM. The explicit 1D-uFDM numerically stability condition is derived in “Methods: Explicit 1D-uFDM numerical stability condition” and tested numerically in Supplementary: Numerical verification of stability Conditions. A fully implicit 1D-uFDM is ill defined (Supplementary: An implicit 1D-uFDM is ill defined); however, a semi-implicit 1D-uFDM and numerical stability condition can be derived (“Methods: Semi-implicit 1D-uFDM solution, Methods: Semi-implicit 1D-uFDM numerical stability condition”). The explicit and semi-implicit 1D-uFDMs solve the 2D diffusion equation in a 1D reduced-dimension model.

In all three illustrative examples below, we are testing the ability of the explicit 1D-uFDM to solve the 2D diffusion equation in a 1D reduced-dimension model. We will also illustrate the accuracy gained by solving the 2D diffusion equation in a 1D reduced-dimension model using the 1D-uFDM, when compared to using the 1D diffusion equation. Thus, the solutions of the 1D-uFDM and 1D-FDM will be compared to the solution on a slice through the centre of the patch in the full system. The 2D solution will be calculated using 2D-FDM.

### Illustrative example 1: Diffusion

**Fig. 2:**
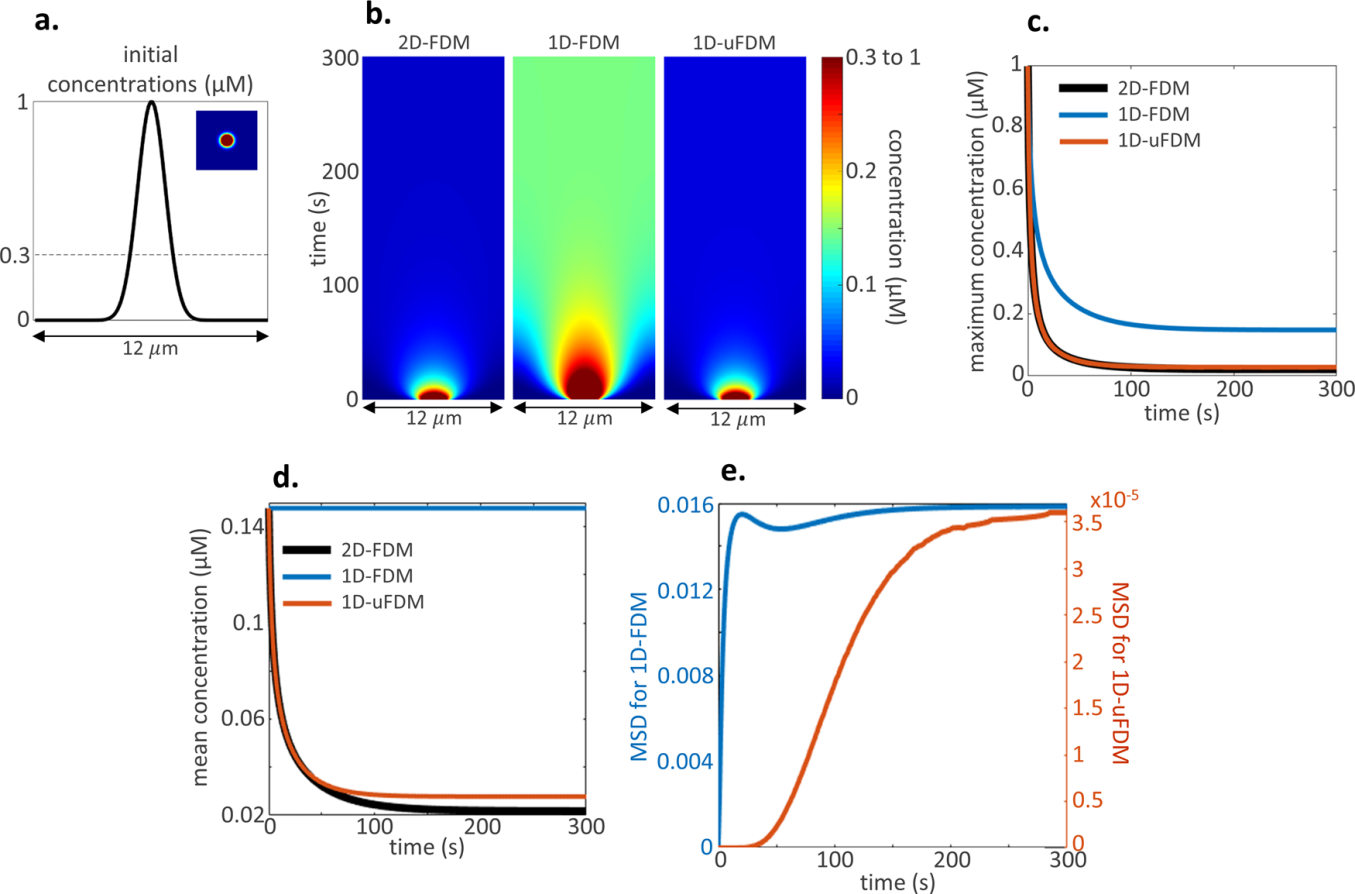
Illustrative example 1: Diffusion initial conditions and results. **a** Initial concentrations for diffusion solutions, ${u}_{1D}\left(x,0\right)={e}^{-{x}^{2}}$ and inset ${u}_{2D}\left(x,y,0\right)={e}^{-\left({x}^{2}+{y}^{2}\right)}$. The dotted line shows the threshold for colorplots (inset and **b**). The threshold was chosen to highlight differences in dynamics and homogeneous steady states in kymographs. **b** Kymographs of the 2D solution along a slice through the centre of the initial patch, and the 1D solutions. **c** Concentration at the centre of the initial patch plotted over time for each solution. In this illustrative example the central concentration is the maximum concentration. **d** Mean concentration in the 1D solutions compared with the mean concentration of the 2D solution along a slice through the centre of the initial patch. **e** Mean squared distance (MSD) between the concentrations along a slice through the centre of the initial patch in the 2D solution and the 1D solutions. 1D-FDM comparison is shown in blue, and 1D-uFDM comparison in red.

First, we considered the diffusion of molecules, $u$, from a concentrated patch on the membrane. The initial concentration profile in the reduced-dimension models (Fig. 2a) was identical to the initial concentration along a slice through the centre of the patch in the 2D system (Fig. 2a, inset). The diffusion equation was solved using the explicit 2D-FDM, 1D-FDM and 1D-uFDM (Eqs. 1–3), diffusion coefficient $D=0.1\,\upmu {\mathrm{m}}^{2}\,{\mathrm{s}}^{-1}$ (“Methods: Illustrative example 1: Parameters). The solutions of $u$ in both 1D reduced-dimension models were compared with the solutions of $u$ along a slice through the centre of the initial patch (see Supplementary: Accuracy of the semi-implicit 1D-uFDM when simulating diffusion for implicit 2D-FDM, 1D-FDM and semi-implicit 1D-uFDM comparisons). Results: The reduced-dimension model using the 1D diffusion equation to describe diffusive movement (1D-FDM, Eq. 2) was quantitatively inaccurate at estimating the concentration on a slice through the centre of the 2D patch (Fig. 2b, c). This is because on the 2D membrane molecules diffuse out of the focal plane resulting in a reduction in the mean concentration of molecules in the central slice (Fig. 2d). However, molecules in the 1D-FDM model are trapped in the focal plane and the mean concentration of molecules remains constant, resulting in a higher homogeneous steady state (Fig. 2b–d). The 1D-uFDM estimates the movement of molecules out of the focal plane producing a more accurate reduced-dimension representation of the full system (Fig. 2b–e). While the 1D-uFDM represents the 2D system well it does contain error (Fig. 2e), in-depth error analysis can be found in Supplementary: Steady state accuracy of 1D-uFDMs and Accuracy dynamics of 1D-uFDMs.

### Illustrative example 2: Florescence recovery after photobleaching

The 1D-uFDM was able to solve the 2D diffusion equation in a 1D reduced-dimension model when calculating molecules diffusing away from a central patch. We went on to ask, how accurate is the 1D-uFDM when calculating the movement of molecules into a central trough. To answer this question we modelled a florescence recovery after photobleaching (FRAP) experiment. FRAP experiments are used to estimate the diffusion coefficient of a fluorescently tagged protein. In a FRAP experiment fluorophores, attached to a protein of interest, are bleached by a laser. The florescence recovery within the bleached area is recorded and used to estimate a diffusion coefficient.

**Fig. 3:**
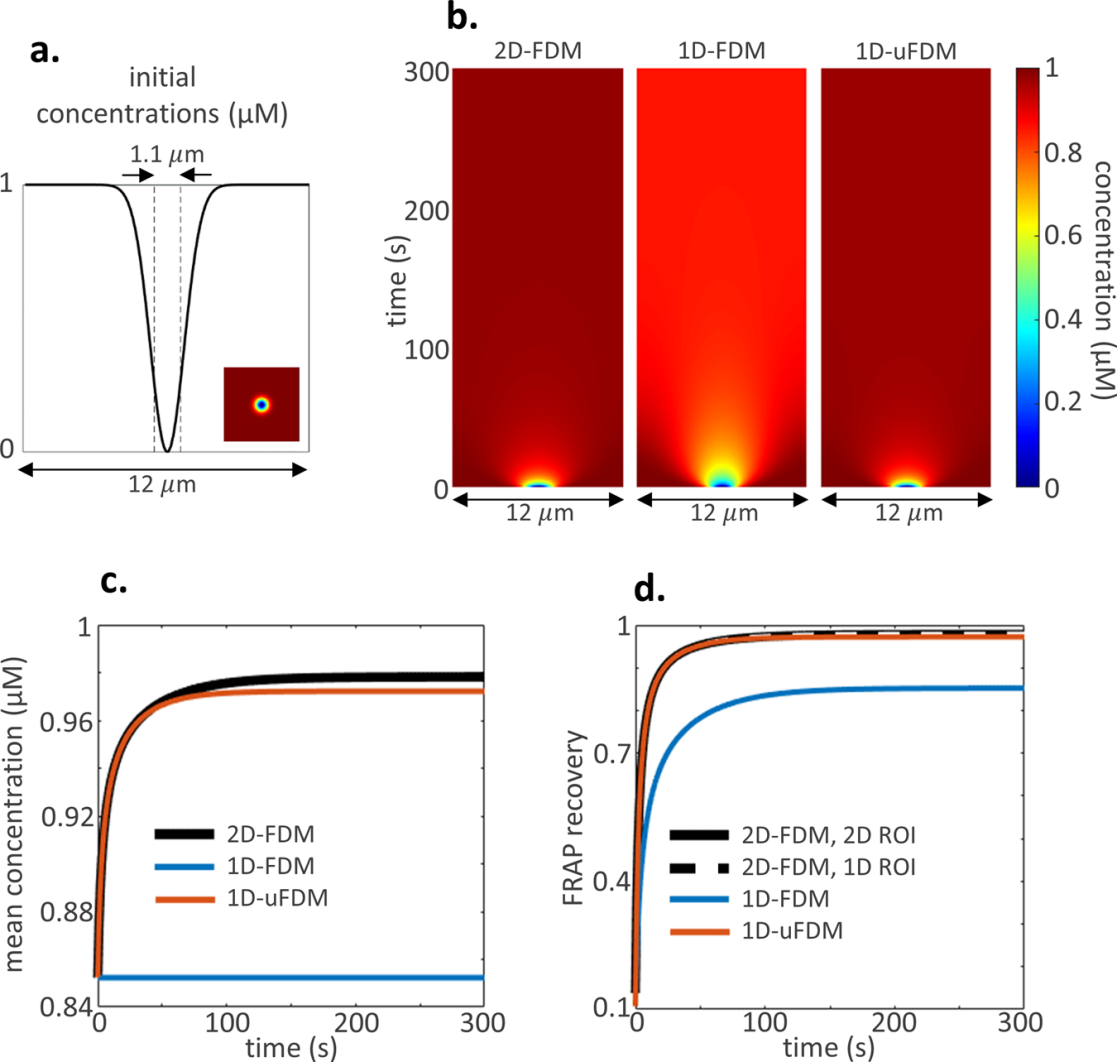
Illustrative example 2: FRAP initial conditions and results. **a** Initial concentrations for FRAP simulations after bleaching with a Gaussian laser, radius $0.55\,\upmu {\mathrm{m}}$^22^. ${u}_{1D}\left(x,0\right)=1-{e}^{-{x}^{2}}$, inset ${u}_{2D}\left(x,y,0\right)=1-{e}^{-\left({x}^{2}+{y}^{2}\right)}$. Dotted lines show the ROI. **b** Kymographs of the 2D solution along a slice through the centre of the bleached area, and the 1D solutions. **c** Mean concentration in the 1D solutions compared with the mean concentration of the 2D solution along a slice through the centre of the bleached area. **d** FRAP recovery curves. The 2D ROI is a circle diameter $1.1\,\upmu {\mathrm{m}}$, 1D ROI a line length $1.1\,\upmu {\mathrm{m}}$.

In our FRAP model the initial fluorophore concentrations were set to reflect a uniformly covered membrane after bleaching with a Gaussian laser^22^ (Fig. 3a). Diffusive movement of the unbleached, fluorescently tagged, proteins was modelled using the 2D-FDM, the 1D-FDM and the 1D-uFDM (Eqs. 1–3), diffusion coefficient $D=0.1\,\upmu {\mathrm{m}}^{2}\,{\mathrm{s}}^{-1}$. Results: In all FRAP solutions fluorescently tagged proteins diffused into the bleached area (Fig. 3b). The 1D-FDM solution had a low homogeneous steady-state concentration when compared to the homogeneous steady state along a slice through the centre of the bleached area in the full system (Fig. 3b). The low steady-state concentration in the 1D-FDM solution can be accounted for by the inability of tagged proteins to move into the focal plane (Fig. 3c). The 1D-uFDM estimated the movement of tagged proteins into the focal plane to give a more accurate representation of the 2D system (Fig. 3b, c). Further accuracy analysis can be found in Supplementary: Accuracy of the 1D-FDM and 1D-uFDM when simulating FRAP.

As FRAP is used to estimate diffusion coefficients we performed FRAP analysis on our simulated FRAP data, “Methods: Illustrative example 2: Parameters and FRAP analysis”. The FRAP recovery curves (Fig. 3d) show the mean florescence recovery within the region of interest (ROI, dotted lines Fig. 3a). The results of FRAP analysis can be found in Table 1. The half time, ${t}_{1/2}$ seconds, is the time needed for the florescence intensity to reach half its maximum recovery. For the 2D system we calculated the half time and estimated the diffusion coefficient, $\widetilde{D}\,\upmu {\mathrm{m}}^{2}\,{\mathrm{s}}^{-1}$, using the full 2D solution and the data along a slice through the centre of the bleached area. Both methods provided comparable results, estimating the diffusion coefficient accurately to one decimal place (Table 1). FRAP analysis on the 1D-FDM estimated the diffusion coefficient to be half its actual value. In order for a 1D-FDM solution to estimate a diffusion coefficient $\widetilde{D}=0.1\,\upmu {\mathrm{m}}^{2}\,{\mathrm{s}}^{-1}$, the actual diffusion coefficient had to be increased to $D=0.22\,\upmu {\mathrm{m}}^{2}\,{\mathrm{s}}^{-1}$ (Table 1). FRAP analysis on the 1D-uFDM solution resulted in an accurate estimation of the diffusion coefficient. Thus, the 1D-uFDM is able to estimate 2D diffusive movement into a trough. Furthermore, these results show that data obtained from membrane FRAP experiments could be used to fit parameters in 1D-uFDM reduced-dimension models.

**Table 1:** Results of FRAP analysis on the 1D and 2D solutions.

$D=0.1\,\upmu {\mathrm{m}}^{2}\,{\mathrm{s}}^{-1}$	ROI	${t}_{1/2}s$	$\tilde{D}$ $\upmu {\mathrm{m}}^{2}\,{\mathrm{s}}^{-1}$
2D-FDM	2D	$2.711$	$0.106$
2D-FDM	1D	$2.801$	$0.103$
1D-FDM	1D	$6.145$	$0.047$
1D-uFDM	1D	$2.678$	$0.108$

$$D=0.22\,\upmu {\mathrm{m}}^{2}\,{\mathrm{s}}^{-1}$$	ROI	$${t}_{1/2}s$$	$$\tilde{D}\;$$$$\upmu {\mathrm{m}}^{2}\,{\mathrm{s}}^{-1}$$
1D-FDM	1D	$2.678$	$0.108$

Half time, $${t}_{1/2}$$ seconds, actual diffusion coefficients, $$D\,\upmu {\mathrm{m}}^{2}\,{\mathrm{s}}^{-1}$$, and estimated diffusion coefficients, $$\tilde{D}\,$$$${{\upmu }}{{\rm{m}}}^{2}\,{{\rm{s}}}^{-1}$$. The 2D ROI is a circle diameter $$1.1\,\upmu {\mathrm{m}}$$, 1D ROI a line length $$1.1\,\upmu {\mathrm{m}}$$.

### Illustrative example 3: Reaction-diffusion dynamics

**Fig. 4:**
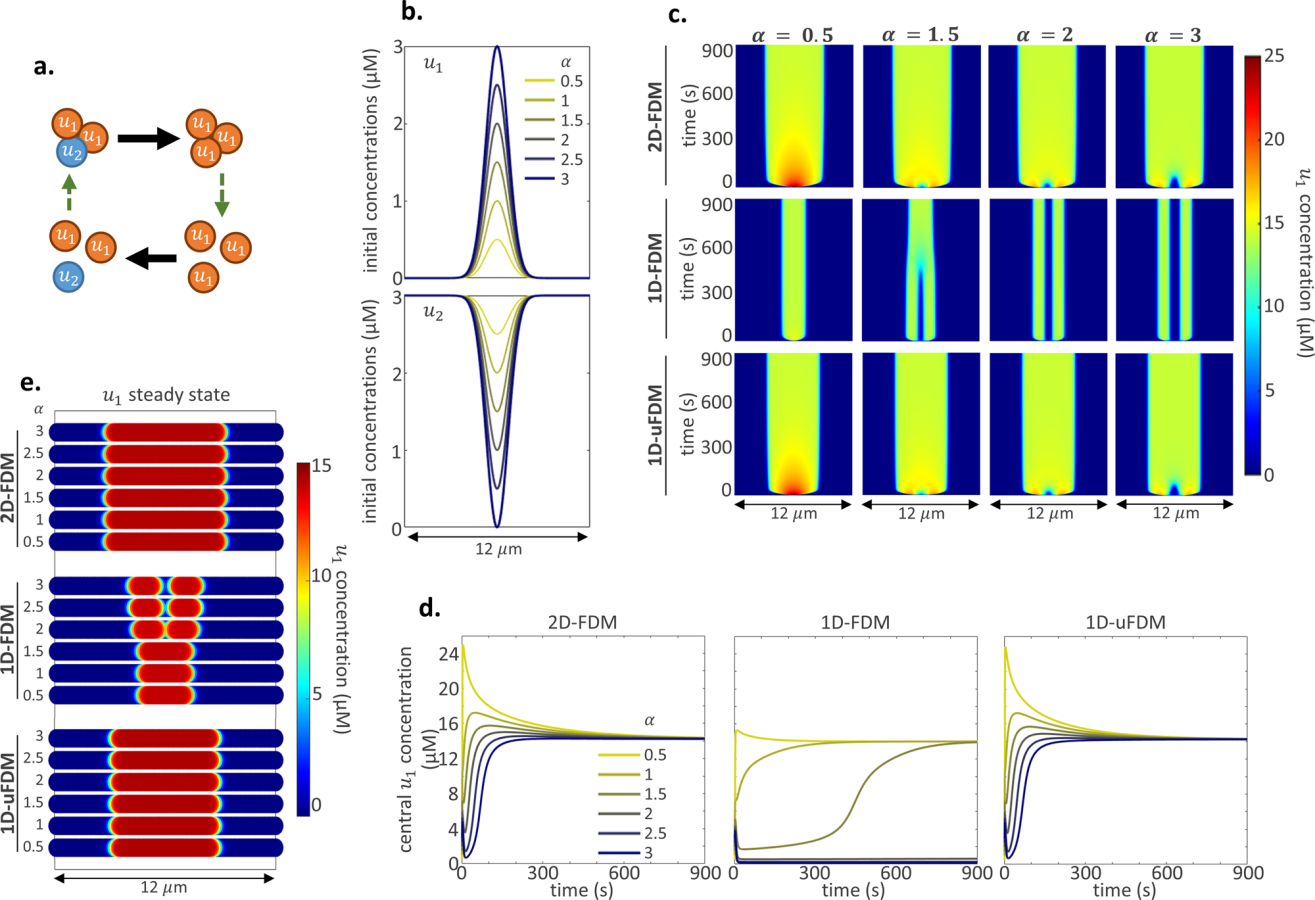
Illustrative example 3: Reaction-diffusion initial conditions and results. **a** Cartoon of the RD model. Black arrows represent reactions and green dashed arrows represent diffusion. Mass is conserved, molecules are only changed, never created or lost. **b** Initial concentrations of ${u}_{1}$ and ${u}_{2}$ for the RD models. $\alpha =[{\mathrm{0.5,1}},\ldots ,3]$ is the maximum concentration of the ${u}_{1}$ peak after the initial signalling pulse. ${u}_{1}\left(x,y,0\right)={\alpha e}^{-\left({x}^{2}+{y}^{2}\right)}$, ${u}_{1}\left(x,0\right)={\alpha e}^{-{x}^{2}}$ and ${u}_{2}=m-{u}_{1}$, where $m$ is the mass of the system, see “Methods: Illustrative example 3: RD parameters and simulations”. **c** Kymographs of the ${u}_{1}$ concentration in the 2D solution along a slice through the centre of the initial signalling pulse, and the 1D solutions. **d** ${u}_{1}$ concentration dynamics at the centre of the initial signalling pulse in the 1D and 2D solutions. **e** Steady-state colorplots of the 2D and 1D ${u}_{1}$ solutions.

Spatial models rarely focus solely on diffusion. We asked to what extent could 1D-uFDM reaction-diffusion (RD) model capture 2D RD dynamics along a slide through the full system’s symmetry. To address this question, a two component, mass conserved, substrate depletion model was used^8^ (Fig. 4a, Methods: Illustrative example 3: RD parameters and simulations).

4 $$\frac{\partial }{\partial t}{u}_{1}={u}_{1}^{2}{u}_{2}-{u}_{1}+\left(0.01\right){\nabla }^{2}{u}_{1}$$

5 $$\frac{\partial }{\partial t}{u}_{2}=-\left({u}_{1}^{2}{u}_{2}-{u}_{1}\right)+{\nabla }^{2}{u}_{2}$$

${\nabla }^{2}$ denotes diffusion, the Laplacian, it is this part of the RD equation which will be estimated by the finite difference methods (Eqs. 1–3). Initial concentrations were set to represent a signalling event which caused a pulse conversion of molecules to ${u}_{1}$ from the ${u}_{2}$ pool (Fig. 4b). After this initial signalling event the dynamics of the system were described by the RD equations. As in previous illustrative examples, the RD equations were solved in the full 2D system and the two 1D reduced-dimension models. Diffusion was solved using the 2D-FDM, the 1D-FDM and the 1D-uFDM. The RD model was used to explore the effect of a progressively stronger initial signalling pulse on the dynamics of each solution. Results: For all solutions, larger initial signalling pulses deplete local ${u}_{2}$ such that the RD positive feedback becomes ineffective (${u}_{1}^{2}{u}_{2}$ is very small) and a ${u}_{1}$ trough is soon formed at the location of the initial pulse (Fig. 4c, d). Similar to the FRAP analysis results, ${u}_{1}$ molecules were replenished more slowly in the 1D-FDM RD solution than in the 2D-FDM and 1D-uFDM RD solutions. Thus, in the 1D-FDM RD solution, as the initial signalling pulse increases, creating a larger trough, movement into the trough becomes insufficient to reach the centre and two narrow peaks are formed (Fig. 4c, e). The 1D-FDM solution is qualitatively different from the 2D-FDM solution for $\alpha \ge 1.5$ (Fig. 4c–e). The 1D-uFDM reproduced all the 2D-FDM RD molecular dynamics through the focal plane (Fig. 4c–e), showing that it can be used to increase the accuracy of reduced-dimension RD models. Further results and accuracy analysis can be found in Supplementary: Reaction-diffusion comparisons and the effect of geometry.

## Methods

### Generating the interpolation mesh in 1D

Consider a 1D reduced-dimension model, reduced from a 2D uniform mesh (Fig. 1a). To solve the 1D-uFDM one must use the concentrations on the 1D mesh, row $J$ of the 2D mesh, to estimate concentrations at mesh points in rows $J\pm 1$ of the 2D mesh (Fig. 1d, f, g). The reduced-dimension model is a ring and so has periodic boundary conditions. Without loss of generality, we will assume the centre of the patch at $i=N/2$. To generate the mesh to be interpolated from re-index the 1D mesh using $n=i-N/2$. The transformed index, to be interpolated from is $n=\left\{\left(1-\frac{N}{2}\right),\left(2-\frac{N}{2}\right),\ldots -{1,0,1},\ldots \left(\frac{N}{2}-1\right),\frac{N}{2}\right\}$. To generate the mesh to be interpolated to use the transformed index to calculate the distance of each point in rows $J\pm 1$ from the centre of the patch using the equation $\sqrt{{\left({\rm{n}}\triangle {\rm{x}}\right)}^{2}+{\triangle {\rm{y}}}^{2}}$ for $n=\left\{\left(1-\frac{N}{2}\right),\left(2-\frac{N}{2}\right),\ldots -{\mathrm{1,0,1}},\ldots \left(\frac{N}{2}-1\right),\frac{N}{2}\right\}$ (Fig. 1g). Then interpolate. For systems with radial symmetry one need only estimate concentrations at a quarter of the phantom points to solve the 1D-uFDM, for example, $n=\left\{{\mathrm{0,1}},\ldots \left(\frac{N}{2}-1\right),\frac{N}{2}\right\}$. The accuracy of estimating concentrations in rows $J\pm 1$ of the 2D mesh, using interpolation, is discussed in Supplementary: Accuracy of estimating concentrations at phantom points using interpolation.

### Explicit 1D-uFDM derivation

The explicit 2D-FDM is

6 $${u}_{i,j}^{\tau +1}={u}_{i,j}^{\tau }+{d}_{x}\left({u}_{i-1,j}^{\tau }-2{u}_{i,j}^{\tau }+{u}_{i+1,j}^{\tau }\right)+{d}_{y}\left({u}_{i,j-1}^{\tau }-2{u}_{i,j}^{\tau }+{u}_{i,j+1}^{\tau }\right)$$

where ${d}_{x}=\frac{\triangle t}{{\triangle x}^{2}}D$ and ${d}_{y}=\frac{\triangle t}{{\triangle y}^{2}}D$. $J$ is the row of mesh points through the centre of the patch, the focal plane. When estimating 2D diffusion, through the centre of the patch, in 1D space, we only have information about row $J$. Thus, we have to estimate the concentrations ${u}_{i,J-1}^{\tau }$ and ${u}_{i,J+1}^{\tau }$. To achieve this we use the property of radial symmetry exhibited by a patch of proteins. Transform the $\triangle x$ mesh points such that the centre of the patch is at mesh point $n=0$ (see section “Methods: Generating the interpolation mesh in 1D”). The property of radial symmetry dictates that, for $k\ge 0$, ${u}_{k,J+1}^{\tau }={u}_{k,J-1}^{\tau }={u}_{-k,J+1}^{\tau }={u}_{-k,J-1}^{\tau }$ (Fig. 1d, f). Denote the estimated concentration ${u}_{n,J\pm 1}^{\tau }$ as ${u}_{\sqrt{{\left(n\triangle x\right)}^{2}+{\triangle y}^{2}}}^{\tau }$ $\forall n=\left\{\left(1-\frac{N}{2}\right),\ldots \frac{N}{2}\right\}$ (Fig. 1g). Substituting the interpolated values of ${u}_{n,J\pm 1}^{\tau }$ into the 2D-FDM equations, and removing the $J$ subscript we get the 1D-uFDM,

7 $${u}_{n}^{\tau +1}={u}_{n}^{\tau }+{d}_{x}\left({u}_{n-1}^{\tau }-2{u}_{n}^{\tau }+{u}_{n+1}^{\tau }\right)+2{d}_{y}\left({u}_{\sqrt{{\left(n\triangle x\right)}^{2}+{\triangle y}^{2}}}^{\tau }-{u}_{n}^{\tau }\right)$$

### Explicit 1D-uFDM solution

To solve the explicit 1D-uFDM we write the 1D-uFDM equation in matrix form

8 $${\underline{u}}^{\tau +1}=A{\underline{u}}^{\tau }+2{d}_{y}{\underline{\tilde{u}}}_{J}^{\tau }$$

where ${\underline{u}}^{\tau }$ denotes the $N$ × $1$ vector of concentrations ${u}_{n}^{\tau }$ on mesh points $n=\left\{\left(1-\frac{N}{2}\right),\ldots \frac{N}{2}\right\}$, ${\underline{\tilde{u}}}_{J}^{\tau }$ denotes the $N$ × $1$ vector of interpolated concentrations ${u}_{\sqrt{{\left(n\triangle x\right)}^{2}+{\triangle y}^{2}}}^{\tau }$, $n=\left\{\left(1-\frac{N}{2}\right),\ldots ,\frac{N}{2}\right\}$, and

9 $$A=\left[\begin{array}{c}1-2\left({d}_{x}+{d}_{y}\right) 	 {d}_{x} 	 	 	 {d}_{x} \\ {d}_{x} 	 1-2\left({d}_{x}+{d}_{y}\right) 	 \qquad {d}_{x} \qquad 	 	 \\ 	 	 \ddots 	 	 \\ 	 	 \qquad {d}_{x} \qquad 	 1-2\left({d}_{x}+{d}_{y}\right) 	 {d}_{x} \\ {d}_{x} 	 	 	 {d}_{x} 	 1-2\left({d}_{x}+{d}_{y}\right) \end{array}\right]$$

a tridiagonal $N$ × $N$ matrix, with periodic boundary conditions. Using interpolation on the matrix form we derive the solution to the explicit 1D-uFDM,

9 $${\underline{u}}^{\tau }={A}^{\tau }{\underline{u}}^{0}+2{d}_{y}\mathop{\sum }\limits_{k=1}^{\tau }{A}^{\tau -k}{\underline{\tilde{u}}}_{J}^{k-1}$$

### Explicit 1D-uFDM numerical stability condition

Let $$\underline{\lambda }$$ denote the vector of eigenvalues for matrix $$A$$. To calculate the stability condition for 1D-uFDM numerical stability recall that the values $${\underline{\tilde{u}}}_{j}^{\tau }$$ are interpolated from $${\underline{u}}^{\tau }$$ at time $$\tau$$, thus, as long as a stable interpolation method is used, the solution will be stable if $$|\lambda |$$ $$\le 1 \; \forall \; \lambda \in \,$$$$\underline{\lambda }$$. Gerschgorin’s circle theorem^21^ states that $$\lambda$$ is bounded by the inequality,

10 $$1-2\left({d}_{x}+{d}_{y}\right)-2{d}_{x}\le \lambda \le 1-2\left({d}_{x}+{d}_{y}\right)+2{d}_{x}$$

which can be simplified to,

11 $$1-4{d}_{x}-2{d}_{y}\le \lambda \le 1-2{d}_{y}$$

Thus, the 1D-uFDM solution will be numerically stable if $1-2{d}_{y}\le 1$ and $-1\le 1-4{d}_{x}-2{d}_{y}$. $1-2{d}_{y}\le 1$ is always satisfied as ${d}_{y} \,> \,0$. The inequality $-1\le 1-4{d}_{x}-2{d}_{y}$ leads to the explicit 1D-uFDM stability condition,

12 $$2{d}_{x}+{d}_{y}\le 1$$

The explicit 1D-uFDM stability condition is numerically verified in Supplementary: Numerical verification of stability conditions.

### Semi-implicit 1D-uFDM: derivation

A fully implicit 1D-uFDM is ill defined as molecular movement through the focal plane is inferred using the concentrations on the focal plane, Supplementary: An implicit 1D-uFDM is ill defined. A semi-implicit numerical solver can be defined in which molecular movement through the focal plane is solved explicitly and molecular movement on the focal plane is solved implicitly. The semi-implicit 2D-FDM equation is

13 $${u}_{i,j}^{\tau +1}={u}_{i,j}^{\tau }+{d}_{x}\left({u}_{i-1,j}^{\tau +1}-2{u}_{i,j}^{\tau +1}+{u}_{i+1,j}^{\tau +1}\right)+{d}_{y}\left({u}_{i,j-1}^{\tau }-2{u}_{i,j}^{\tau }+{u}_{i,j+1}^{\tau }\right)$$

Using the same reasoning used for the derivation of the explicit 1D-uFDM, the semi-implicit 1D-uFDM is

14 $${u}_{n}^{\tau +1}={u}_{n}^{\tau }+{d}_{x}\left({u}_{n-1}^{\tau +1}-2{u}_{n}^{\tau +1}+{u}_{n+1}^{\tau +1}\right)+2{d}_{y}\left({u}_{\sqrt{{\left(n\triangle x\right)}^{2}+{\triangle y}^{2}}}^{\tau }-{u}_{n}^{\tau }\right)$$

Note, the semi-implicit 1D-uFDM scheme mirrors the 1D Crank-Nicolson method which converges and is unconditionally stable^21^. We will show that the same is not true for the semi-implicit 1D-uFDM. However, the semi-implicit 1D-uFDM has less strict numerical stability conditions than the explicit 1D-uFDM.

### Semi-implicit 1D-uFDM: solution

To solve the semi-implicit 1D-uFDM we write it in matrix form,

15 $${\underline{u}}^{\tau +1}={C}^{-1}\Big(\Big(1-2{d}_{y}\Big){\underline{u}}^{\tau }+2{d}_{y}{\underline{\tilde{u}}}_{J}^{\tau }\Big)$$

where

$$C=\left[\begin{array}{c}1+2{d}_{x} 	 -{d}_{x} 	 	 	-{d}_{x}\\ -{d}_{x} 	 1+2{d}_{x} 	 \;\;\; -{d}_{x} \;\;\; 	 	 \\ 	 	 \ddots 	 	 \\ 	 	 \;\;\; -{d}_{x} \;\;\; 	1+2{d}_{x} 	 -{d}_{x}\\ -{d}_{x} 	 	 	 -{d}_{x} 	 1+2{d}_{x} \end{array}\right]$$

a tridiagonal $N$ × $N$ matrix, with periodic boundary conditions. Again, using interpolation on the matrix form of the equation we derive the solution to the semi-implicit 1D-uFDM,

16 $${\underline{u}}^{k+1}={C}^{-(k+1)}{\Big(1-2{d}_{y}\Big)}^{k+1}{\underline{u}}^{0}+2{d}_{y}\mathop{\sum }\limits_{l=1}^{k}{\Big(1-2{d}_{y}\Big)}^{l-1}{C}^{-1}{\underline{\tilde{u}}}_{J}^{k-l}$$

### Semi-implicit 1D-uFDM: numerical stability condition

Let $\underline{\lambda }$ be the vector of eigenvalues for $C$. For the solution of the semi-implicit 1D-uFDM to be stable two inequalities must hold, $\left|1/\lambda \right|\le 1$ and $\left|1-2{d}_{y}\right|\le 1$. The first inequality $1\le \left|\lambda \right|$ can be investigated using Gerschgorin’s circle theorem^21^. Gerschgorin’s circle theorem states that

17 $$1+2{d}_{x}-2{d}_{x}\le \lambda \le 1+2{d}_{x}+2{d}_{x}$$

which can be simplified to

18 $$1\le \lambda \le 1+4{d}_{x}$$

Thus, the inequality $1\le |\lambda |$ is always satisfied. The second inequality $|1-2{d}_{y}|\le 1$ expands to $-1\le 1-2{d}_{y}\le 1$. $1-2{d}_{y}\le 1$ as ${d}_{y}\ge 0$. $-1\le 1-2{d}_{y}$ leads to the semi-implicit 1D-uFDM stability condition,

19 $${d}_{y}\le 1$$

The semi-implicit 1D-uFDM stability condition is numerically verified in Supplementary: Numerical verification of stability conditions.

### Illustrative example 1: Parameters

For results shown in Fig. 2b–e, to enable comparisons between the different numerical method solutions, the explicit 2D-FDM, 1D-FDM and 1D-uFDM were all solved using the same parameters: $D=0.1\,\upmu {\mathrm{m}}^{2}\,{\mathrm{s}}^{-1}$, $\Delta x=\Delta y=0.1\,\upmu {\mathrm{m}}$, $\Delta t=0.01{\mathrm{s}}$. These values were chosen to satisfy the numerical stability conditions of all three numerical methods. The explicit 1D-uFDM accuracy analysis found in Supplementary: Steady state accuracy of 1D-uFDMs and Accuracy dynamics of 1D-uFDMs was also taken into consideration.

### Illustrative Example 2: Parameters and FRAP analysis

For the comparative FRAP analysis, $D=0.1\,\upmu {\mathrm{m}}^{2}\,{\mathrm{s}}^{-1}$, $\Delta x=\Delta y=0.1\,\upmu {\mathrm{m}}$, $\Delta t=0.01{\mathrm{s}}$ for all solutions. The 2D ROI used to calculate the FRAP recovery curve was a circle radius $0.55\,\upmu {\mathrm{m}}$ and the 1D ROI a line $1.1\,\upmu {\mathrm{m}}$ in length (Fig. 3a). Both ROIs were placed in the centre of the bleached region. ${t}_{1/2}$ was calculated using linear interpolation on the FRAP recovery curve (Fig. 3d). To estimate the diffusion coefficient the equation,

20 $$\tilde{D}=\frac{{r}_{n}^{2}+{r}_{e}^{2}}{8{t}_{\frac{1}{2}}}$$

was used^22^, where ${r}_{e}$ represents the value of the effective radius and ${r}_{n}$ the laser radius. For the FRAP simulations, ${r}_{e}=\surd 2$ and ${r}_{n}=0.55$.

### Illustrative example 3: RD parameters and simulations

Symmetry breaking parameters, and a mass sufficient for the system to exhibit saturation due to substrate depletion ($m=3$) were chosen^8^. The mass of the system was defined as the mean concentration of molecules,

21 $$m=\left\langle {u}_{1}\right\rangle +\left\langle {u}_{2}\right\rangle$$

where, for $$k=\left\{{1,2}\right\}$$,

22 $$\left\langle {u}_{k}\right\rangle =\frac{1}{{NM}}\mathop{\sum }_{i=1}^{N}\mathop{\sum }_{j=1}^{M}{{u}_{k}}_{\left(i,j\right)}$$

for the 2D case, and,

23 $$\left\langle {u}_{k}\right\rangle =\frac{1}{N}\mathop{\sum }_{i=1}^{N}{{u}_{k}}_{\left(i\right)}$$

for 1D.

$\Delta x=\Delta y=0.1\,\upmu {\mathrm{m}}$. Solving the RD equations was a done using a two-step process: reactions were solved using Euler’s method at time steps $\Delta {t}_{R}=1{{\times}}{10}^{-5}{\mathrm{s}}$ for all simulations, diffusion was solved using explicit 2D-FDM, 1D-FDM and 1D-uFDM at time steps $\Delta {t}_{D}=0.002{\mathrm{s}}$ to ensure numerical stability of the explicit 2D-FDM.

### Reporting summary

Further information on research design is available in the Nature Research Reporting Summary linked to this article.

## Supplementary information

Supplementary Information

Reporting Summary

## Data Availability

The underlying data are present in the manuscript itself and its supplementary information file. Any other remaining information can be obtained from the corresponding author upon reasonable request.

## Code availability

All codes, plotters and raw figure files used in the generation of Illustrative Examples 1–3 are available through Zenodo^23^ and GitHub^24^.
