# Supplementary material for: Describing the movement of molecules in reduced-dimension models
Source: Commun Biol. 2021 Jun 7;4:689. doi: 10.1038/s42003-021-02200-3 (PMC8184792; doi:10.1038/s42003-021-02200-3)
Supplement: Supplementary file 1 — Supplementary Information [file 42003_2021_2200_MOESM1_ESM.pdf]

Describing the movement of molecules in reduced-dimension models.

Natasha S. Savage.

University of Liverpool, Liverpool, UK.

Email: nsavage@liverpool.ac.uk

Supplementary Information

Accuracy of estimating concentrations at phantom points using interpolation.

Numerical solutions within this Brief Communication were performed in MATLAB. Spline interpolation was found to be the most accurate interpolation method in MATLAB for the concentration profiles investigated here (data not shown). However, if a concentration profile approaches zero with a steep gradient spline interpolation can return a small negative concentration. Any negative concentrations resulting from MATLAB spline interpolation were set to zero.

To investigate the accuracy of estimating concentrations at phantom mesh-points (those in rows $J \pm 1$) using MATLAB spline interpolation, 2D concentration profiles were generated and the actual concentrations on rows $J \pm 1$ were compared with the estimated concentrations, which had been interpolated from the 1D concentration profile (Main Methods). To quantify interpolation accuracy the sum squared distance (SSD) between the interpolated and actual concentration profiles was calculated.

The concentration profiles used to investigate the accuracy of estimating concentrations at phantom points can be seen in Supplementary Figure 1. To better understand the influence of Δx and Δy on the estimation of phantom Point concentrations, a range of Δx and Δy values, between $0.001\,\mu m$ and $0.1\,\mu m$, was used for each concentration profile. As expected, increasing Δx decreases the accuracy of the interpolation (Supplementary Figure 2, column 1). Comparing columns 1 and 2 of Supplementary Figure 2 we see that the error around the concentration peak, in the central 10% of the mesh, accounts for most of the interpolation error. Indeed, the greater the curvature of the concentration profile the greater the interpolation error. In order to estimate concentrations at phantom points on the special boundaries one must interpolate outside the data (Supplementary Figure 5b). Supplementary Figure 2, column 3, shows that, as we may expect, the greater Δy the greater the error in estimation outside the boundary.

Supplementary Table 1 shows the maximum and minimum SSD when estimating the phantom points of all the concentration profiles shown in Supplementary Figures 1a, c, and e. The estimation error is small for small Δx and Δy values (Supplementary Figure 2 and Supplementary Table 1). Decreasing Δx increases computation time, to reduce estimation error for larger Δx a small Δy should be chosen (Supplementary Figure 2). The choices of Δx and Δy should be chosen to satisfy the stability conditions of the numerical method (Main Methods).

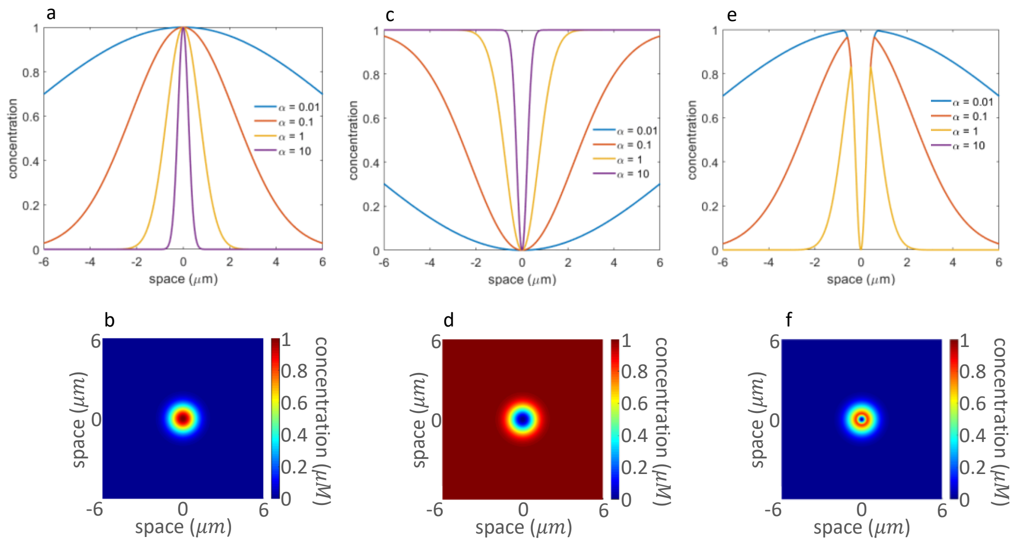

Supplementary Figure 1: Concentration profiles used to investigate the accuracy of estimating concentrations at phantom points. (a) 1D concentration profiles described by the equation, $\boldsymbol{u} = \boldsymbol{e}^{-\alpha x^2}$. **(b)** Example of the 2D system before dimension reduction reduced, $\boldsymbol{\alpha} = \mathbf{1}$, $\boldsymbol{u} = \boldsymbol{e}^{-(x^2+y^2)}$. **(c)** 1D concentration profiles described by the equation, $\boldsymbol{u} = \mathbf{1} - \boldsymbol{e}^{-\alpha x^2}$. **(d)** Example of the 2D system before dimension reduction, $\boldsymbol{\alpha} = \mathbf{1}$, $\boldsymbol{u} = \mathbf{1} - \boldsymbol{e}^{-(x^2+y^2)}$. **(e)** 1D concentration profiles described by the equation, $\boldsymbol{u} = \mathbf{min}\left(\boldsymbol{e}^{-\alpha_1 x^2}, \mathbf{1} - \boldsymbol{e}^{-\alpha_2 x^2}\right)$. $\boldsymbol{\alpha_2} = \mathbf{10}$ for all curves. $\boldsymbol{\alpha_1} = \mathbf{1}, \mathbf{0.1}, \mathbf{0.01}$ for the yellow, red, blue curve, respectively. **(f)** Example of the 2D system before dimension reduction, $\boldsymbol{\alpha_1} = \mathbf{1}$, $\boldsymbol{u} = \mathbf{min}\left(\boldsymbol{e}^{-(x^2+y^2)}, \mathbf{1} - \boldsymbol{e}^{-\mathbf{10}(x^2+y^2)}\right)$.

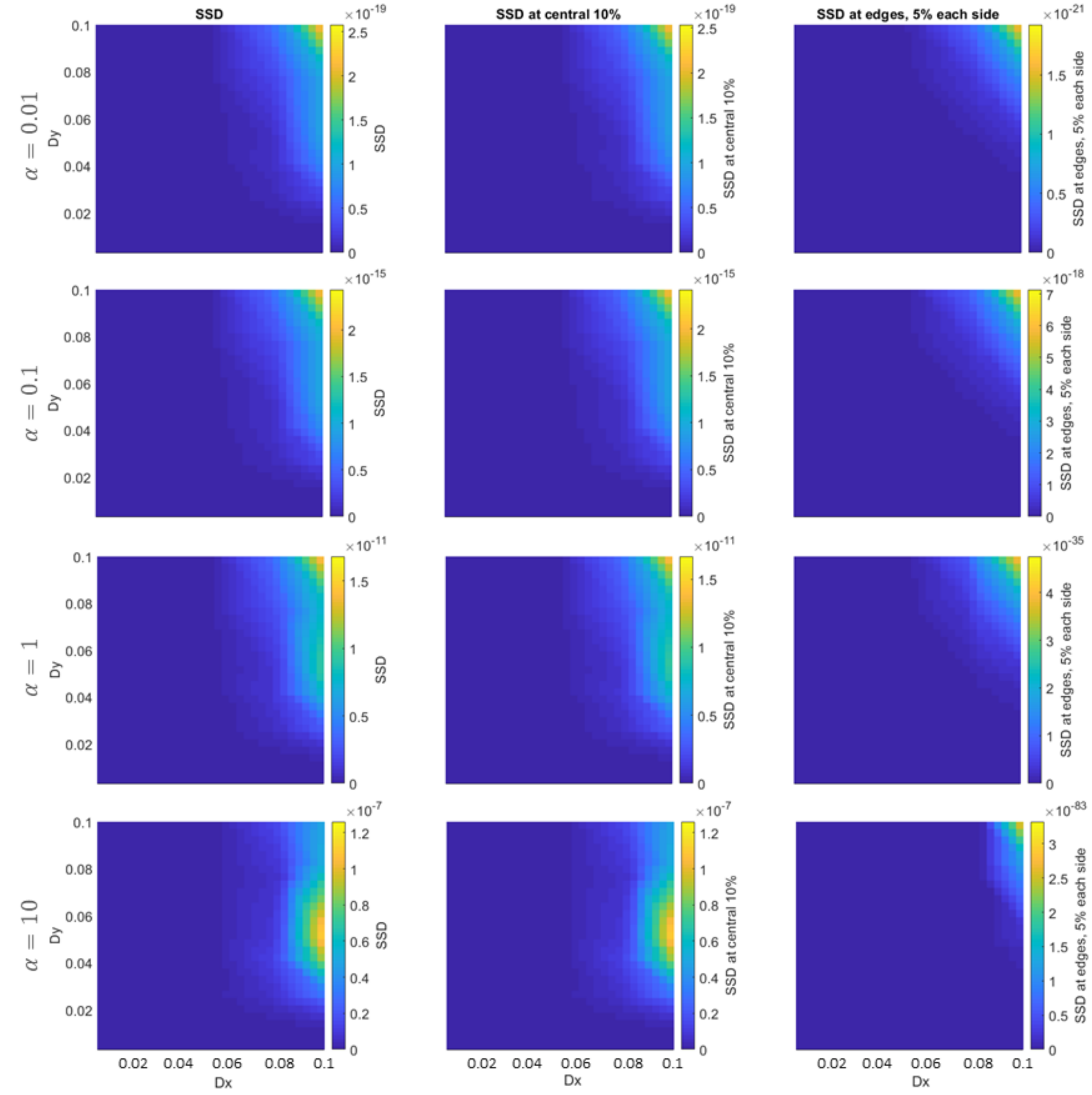

Supplementary Figure 2: SSD between estimated concentrations at phantom points and actual concentrations in the 2D System. The analysis was performed on the profiles, $\boldsymbol{u_{1D}} = \boldsymbol{e}^{-\alpha x^2}$ and $\boldsymbol{u_{2D}} = \boldsymbol{e}^{-\alpha(x^2+y^2)}$ (Supplementary Figures 1a and b).

Supplementary Table 1: Minimum and maximum SSDs between estimated concentrations at phantom points and actual concentrations in the 2D System. Profiles analyzed are those in Supplementary Figures 1a, c and e. Minimum SSDs shown in blue text, maximum in black.

Supplementary Figure	1a			1b			1c				
α	whole	central	edges	whole	centre	edges	α_1	α_2	whole	centre	edges
0.01	$9x10^{-30}$<br>$3x10^{-19}$	$1x10^{-30}$<br>$3x10^{-19}$	$3x10^{-31}$<br>$2x10^{-21}$	$7x10^{-30}$<br>$3x10^{-19}$	$8x10^{-31}$<br>$3x10^{-19}$	$2x10^{-31}$<br>$2x10^{-21}$	10	0.01	$6x10^{-16}$<br>$1x10^{-7}$	$3x10^{-23}$<br>$1x10^{-7}$	$3x10^{-31}$<br>$2x10^{-21}$
0.1	$3x10^{-29}$<br>$2x10^{-15}$	$4x10^{-30}$<br>$2x10^{-15}$	$5x10^{-32}$<br>$7x10^{-18}$	$5x10^{-29}$<br>$2x10^{-15}$	$3x10^{-30}$<br>$2x10^{-15}$	$4x10^{-31}$<br>$7x10^{-18}$	10	0.1	$3x10^{-14}$<br>$6x10^{-6}$	$3x10^{-14}$<br>$6x10^{-6}$	$5x10^{-32}$<br>$7x10^{-18}$
1	$3x10^{-27}$<br>$2x10^{-11}$	$3x10^{-27}$<br>$2x10^{-11}$	$2x10^{-53}$<br>$5x10^{-35}$	$3x10^{-27}$<br>$2x10^{-11}$	$3x10^{-27}$<br>$2x10^{-11}$	0<br>$3x10^{-30}$	10	1	$9x10^{-13}$<br>$2x10^{-4}$	$9x10^{-13}$<br>$2x10^{-4}$	$2x10^{-53}$<br>$1x10^{-35}$
10	$3x10^{-23}$<br>$1x10^{-7}$	$3x10^{-23}$<br>$1x10^{-7}$	$2x10^{-275}$<br>$3x10^{-83}$	$3x10^{-23}$<br>$1x10^{-7}$	$3x10^{-23}$<br>$1x10^{-7}$	0<br>0					

Numerical verification of stability Conditions.

The explicit and semi-implicit 1D-uFDM numerical stability conditions were tested numerically. Recall, $D\frac{\Delta t}{\Delta x^2} = d_x$ and $D\frac{\Delta t}{\Delta y^2} = d_y$. Consider first the explicit 1D-uFDM stability condition in terms of Δx, Δy, Δt and D, $2D\frac{\Delta t}{\Delta x^2} + D\frac{\Delta t}{\Delta y^2} \leq 1$. For $\beta \geq 0$ we would expect a numerical solution with $2D\frac{\Delta t}{\Delta x^2} + D\frac{\Delta t}{\Delta y^2} = 1 + \beta$ to be numerically unstable and a numerical solution with $2D\frac{\Delta t}{\Delta x^2} + D\frac{\Delta t}{\Delta y^2} = 1 - \beta$ to be numerically stable. To test the explicit 1D-uFDM stability condition Δx, Δy and D were set and $\Delta t_{\pm}$ was calculated using the formula,

$$2D\frac{\Delta t_{\pm}}{\Delta x^2} + D\frac{\Delta t_{\pm}}{\Delta y^2} = 1 \pm \beta$$

$$\Delta t_{\pm} = \frac{1 \pm \beta}{\mathrm{D}}\left(\frac{\Delta x^2 \Delta y^2}{2\Delta y^2 + \Delta x^2}\right)$$

Numerical solutions were run for $\Delta x = 0.01, 0.02, \ldots, 0.1\ \mu m$, $\Delta y = 0.01, 0.02, \ldots, 0.1\ \mu m$, $D = 0.1\ \mu m^2 s^{-1}$ and $\Delta t = \Delta t_{\pm}\ s$, $\beta = 1x10^{-4}, 1x10^{-3}, 1x10^{-2}$. The initial condition for each simulation was $u(x,t) = u(x,0) = e^{-x^2}$ for $x \in [0,12]\ \mu m$ (Supplementary Figure 1a, $\alpha = 1$). Numerical solutions were calculated for one hour, or until they crashed as a result of numerical instability. Supplementary Figure 3a shows the stability boundaries being tested. Supplementary Table 2 shows the smallest values of β, out of those tested, for which numerical solutions with Δt_+ were numerically unstable and Δt_- were numerically stable. For the values of $\Delta x, \Delta y$ in the blue cells of Supplementary Table 2, Δt_- simulations with $\beta = 1x10^{-4}, 1x10^{-3}$ were numerically unstable. To understand how far from the stability boundary the blue celled values in Supplementary Table 2 were, the $\Delta t_{\pm}$ equation was rearranged such that $\Delta t_{\pm} = \Delta t \pm \beta \Delta t$, where $\Delta t = \frac{1}{\mathrm{D}}\left(\frac{\Delta x^2 \Delta y^2}{2\Delta y^2 + \Delta x^2}\right)$. The values $\Delta t \pm (1x10^{-2})\Delta t$ are shown in Supplementary Table 3.

The semi-implicit 1D-uFDM stability condition was tested numerically using the same methodology, with $\Delta t_{\pm} = \frac{1 \pm \beta}{\mathrm{D}}\Delta y^2$ (Supplementary Figure 3b, Supplementary Table 4). Simulations with Δt_+ were numerically unstable and Δt_- were numerically stable for all Δx, Δy simulated and $\beta = 1x10^{-4}$.

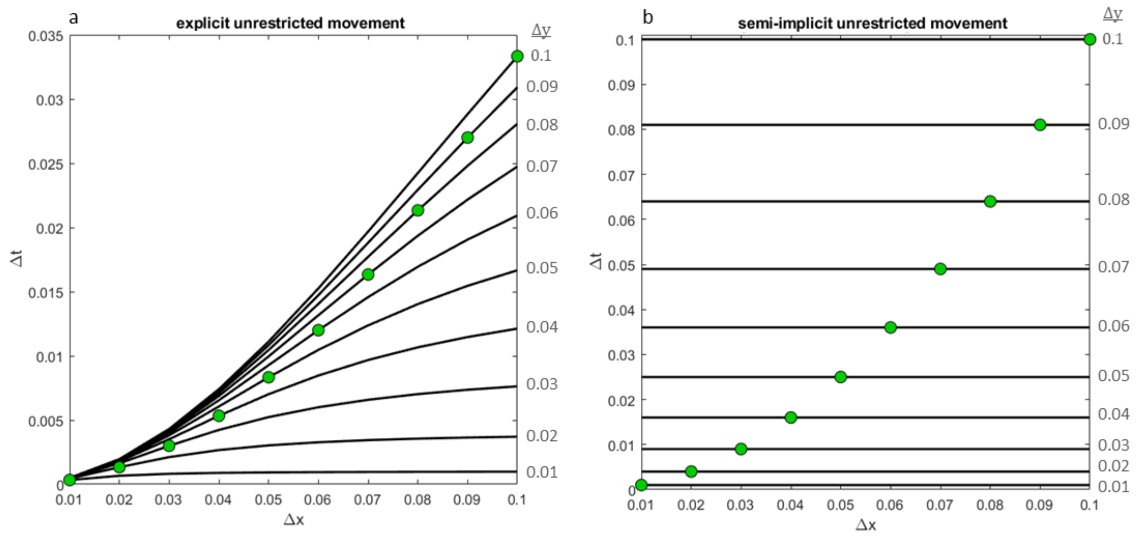

Supplementary Figure 3: Numerical stability boundaries. Black lines show the numerical stability boundaries for $\Delta \boldsymbol{y} = \mathbf{0.01}, \mathbf{0.02}, \ldots, \mathbf{0.1}\ \boldsymbol{\mu m}$. $\boldsymbol{D} = \mathbf{0.1}\ \boldsymbol{\mu m^2 s^{-1}}$ Values of $\Delta \boldsymbol{t}$ on or below the black lines are numerically stable. Green dots show the stability boundary when $\Delta \boldsymbol{x} = \Delta \boldsymbol{y}$. Note, the $\Delta \boldsymbol{t}$ axis have different maxima on each graph. **(a)** Boundaries for numerical stability in the explicit 1D-uFDM are given by the equation, $\Delta \boldsymbol{t} = \frac{1}{D}\left(\frac{\Delta x^2 \Delta y^2}{2\Delta y^2 + \Delta x^2}\right)\ \boldsymbol{s}$. **(b)** Boundaries for numerical stability in the semi-implicit 1D-uFDM are given by the equation, $\Delta \boldsymbol{t} = \frac{\Delta y^2}{D}\ \boldsymbol{s}$.

Supplementary Table 2: Results of the numerical test of the explicit 1D-uFDM stability condition. Smallest values of $\boldsymbol{\beta}$ out of those tested ($\boldsymbol{\beta} = \mathbf{1x10^{-4}}, \mathbf{1x10^{-3}}, \mathbf{1x10^{-2}}$) for which simulations with $\Delta \boldsymbol{t}_+$ were unstable and $\Delta \boldsymbol{t}_-$ were stable. $\boldsymbol{D} = \mathbf{0.1}\ \boldsymbol{\mu m^2 s^{-1}}$. Blue cells indicate the values of $\Delta \boldsymbol{x}, \Delta \boldsymbol{y}$ for which $\Delta \boldsymbol{t}_-$ simulations with $\boldsymbol{\beta} = \mathbf{1x10^{-4}}, \mathbf{1x10^{-3}}$ were numerically unstable.

		Δx									
		**0.01**	**0.02**	**0.03**	**0.04**	**0.05**	**0.06**	**0.07**	**0.08**	**0.09**	**0.1**
Δy	**0.01**	0.0001	0.0001	0.0001	0.0001	0.0001	0.0001	0.0001	0.0001	0.0001	0.0001
	**0.02**	0.0001	0.0001	0.0001	0.0001	0.0001	0.0001	0.0001	0.0001	0.0001	0.0001
	**0.03**	0.01	0.0001	0.0001	0.0001	0.0001	0.0001	0.0001	0.0001	0.0001	0.0001
	**0.04**	0.01	0.0001	0.0001	0.0001	0.0001	0.0001	0.0001	0.0001	0.0001	0.0001
	**0.05**	0.01	0.0001	0.0001	0.0001	0.0001	0.0001	0.0001	0.0001	0.0001	0.0001
	**0.06**	0.01	0.01	0.0001	0.0001	0.0001	0.0001	0.0001	0.0001	0.0001	0.0001
	**0.07**	0.01	0.01	0.0001	0.0001	0.0001	0.0001	0.0001	0.0001	0.0001	0.0001
	**0.08**	0.01	0.01	0.0001	0.0001	0.0001	0.0001	0.0001	0.0001	0.0001	0.0001
	**0.09**	0.01	0.01	0.01	0.0001	0.0001	0.0001	0.0001	0.0001	0.0001	0.0001
	**0.1**	0.01	0.01	0.01	0.0001	0.0001	0.0001	0.0001	0.0001	0.0001	0.0001

Supplementary Table 3: Results of the numerical test of the explicit 1D-uFDM stability condition. $\Delta t_{\pm} = \boldsymbol{\Delta t} \pm \boldsymbol{\beta \Delta t}$ for $\boldsymbol{\beta} = \mathbf{0.01}$.

		Δx		
		**0.01**	**0.02**	**0.03**
Δy	**0.01**			
	**0.02**			
	**0.03**	$4.7\text{x}10^{-4} \pm 4.7\text{x}10^{-6}$		
	**0.04**	$4.8\text{x}10^{-4} \pm 4.8\text{x}10^{-6}$		
	**0.05**	$4.9\text{x}10^{-4} \pm 4.9\text{x}10^{-6}$		
	**0.06**	$4.9\text{x}10^{-4} \pm 4.9\text{x}10^{-6}$	$1.9\text{x}10^{-3} \pm 1.9\text{x}10^{-5}$	
	**0.07**	$4.9\text{x}10^{-4} \pm 4.9\text{x}10^{-6}$	$1.9\text{x}10^{-3} \pm 1.9\text{x}10^{-5}$	
	**0.08**	$5.0\text{x}10^{-4} \pm 5.0\text{x}10^{-6}$	$1.9\text{x}10^{-3} \pm 1.9\text{x}10^{-5}$	
	**0.09**	$5.0\text{x}10^{-4} \pm 5.0\text{x}10^{-6}$	$2.0\text{x}10^{-3} \pm 2.0\text{x}10^{-5}$	$4.2\text{x}10^{-3} \pm 4.2\text{x}10^{-5}$
	**0.1**	$5.0\text{x}10^{-4} \pm 5.0\text{x}10^{-6}$	$2.0\text{x}10^{-3} \pm 2.0\text{x}10^{-5}$	$4.3\text{x}10^{-3} \pm 4.3\text{x}10^{-5}$

Supplementary Table 4: Results of the numerical test of the semi-implicit 1D-uFDM stability condition. Smallest values of $\boldsymbol{\beta}$ out of those tested ($\boldsymbol{\beta} = \mathbf{1x10^{-4}}, \mathbf{1x10^{-3}}, \mathbf{1x10^{-2}}$) for which simulations with $\Delta \boldsymbol{t}_{+}$ were unstable and $\Delta \boldsymbol{t}_{-}$ were stable. $\boldsymbol{D} = \mathbf{0.1}\ \boldsymbol{\mu m^2 s^{-1}}$.

		Δx									
		**0.01**	**0.02**	**0.03**	**0.04**	**0.05**	**0.06**	**0.07**	**0.08**	**0.09**	**0.1**
Δy	**0.01**	0.0001	0.0001	0.0001	0.0001	0.0001	0.0001	0.0001	0.0001	0.0001	0.0001
	**0.02**	0.0001	0.0001	0.0001	0.0001	0.0001	0.0001	0.0001	0.0001	0.0001	0.0001
	**0.03**	0.0001	0.0001	0.0001	0.0001	0.0001	0.0001	0.0001	0.0001	0.0001	0.0001
	**0.04**	0.0001	0.0001	0.0001	0.0001	0.0001	0.0001	0.0001	0.0001	0.0001	0.0001
	**0.05**	0.0001	0.0001	0.0001	0.0001	0.0001	0.0001	0.0001	0.0001	0.0001	0.0001
	**0.06**	0.0001	0.0001	0.0001	0.0001	0.0001	0.0001	0.0001	0.0001	0.0001	0.0001
	**0.07**	0.0001	0.0001	0.0001	0.0001	0.0001	0.0001	0.0001	0.0001	0.0001	0.0001
	**0.08**	0.0001	0.0001	0.0001	0.0001	0.0001	0.0001	0.0001	0.0001	0.0001	0.0001
	**0.09**	0.0001	0.0001	0.0001	0.0001	0.0001	0.0001	0.0001	0.0001	0.0001	0.0001
	**0.1**	0.0001	0.0001	0.0001	0.0001	0.0001	0.0001	0.0001	0.0001	0.0001	0.0001

An implicit 1D-uFDM is ill defined.

The implicit 2D-FDM is,

$$u_{i,j}^{\tau+1} = u_{i,j}^{\tau} + \frac{\Delta t}{\Delta x^2} D\left(u_{i-1,j}^{\tau+1} - 2u_{i,j}^{\tau+1} + u_{i+1,j}^{\tau+1}\right) + \frac{\Delta t}{\Delta y^2} D\left(u_{i,j-1}^{\tau+1} - 2u_{i,j}^{\tau+1} + u_{i,j+1}^{\tau+1}\right)$$

Using the same reasoning as for the derivation of the explicit 1D-uFDM, the implicit 1D-uFDM is,

$$u_n^{\tau+1} = u_n^{\tau} + \frac{\Delta t}{\Delta x^2} D(u_{n-1}^{\tau+1} - 2u_n^{\tau+1} + u_{n+1}^{\tau+1}) + 2\frac{\Delta t}{\Delta y^2} D\left(u_{\sqrt{(n\Delta x)^2+\Delta y^2}}^{\tau+1} - u_n^{\tau+1}\right)$$

The vector form of the implicit 1D-uFDM is,

$$\underline{u}^{\tau} = B\underline{u}^{\tau+1} + 2d_y\underline{\tilde{u}}_J^{\tau+1}$$

$$B = \begin{bmatrix} 1+2(d_x+d_y) & -d_x & & & -d_x \\ -d_x & 1+2(d_x+d_y) & & & \\ & & \ddots & & \\ & & & 1+2(d_x+d_y) & -d_x \\ -d_x & & & -d_x & 1+2(d_x+d_y) \end{bmatrix}$$

a tridiagonal NxN matrix, with periodic boundary conditions.

The solution to the implicit 1D-uFDM is,

$$\underline{u}^{\tau+1} = B^{-1}\left(\underline{u}^{\tau} - 2d_y\underline{\tilde{u}}_J^{\tau+1}\right)$$

However, the values of $\underline{u}^{\tau+1}$ needed to interpolate the values $\underline{\tilde{u}}_J^{\tau+1}$, and so $\underline{\tilde{u}}_J^{\tau+1}$ in the above equation are not defined.

Accuracy of the semi-implicit 1D-uFDM when simulating diffusion.

Fig. 2B-E shows the comparisons between the explicit 2D-FDM, 1D-FDM and 1D-uFDM when modelling diffusion in the full and reduced-dimension models. Supplementary Figure 7 shows the same investigation, but this time comparing the results of the explicit 2D-FDM, 1D-FDM and semi-implicit 1D-uFDM. $D = 0.1\ \mu m^2 s^{-1}$. A set of Δx, Δy and Δt values were chosen that gave an 'improved' steady state accuracy with a fairly small initial MSD peak, namely $\Delta x = \Delta y = 0.1\ \mu m$, $\Delta t = 0.1\ s$ (Supplementary Figure 6f). As expected setting $\Delta t = 0.01\ s$, the same as Δt in the explicit comparison, gave a semi-implicit curve comparable to the explicit MSD curve (compare Supplementary Figure 7b with Fig. 2E).

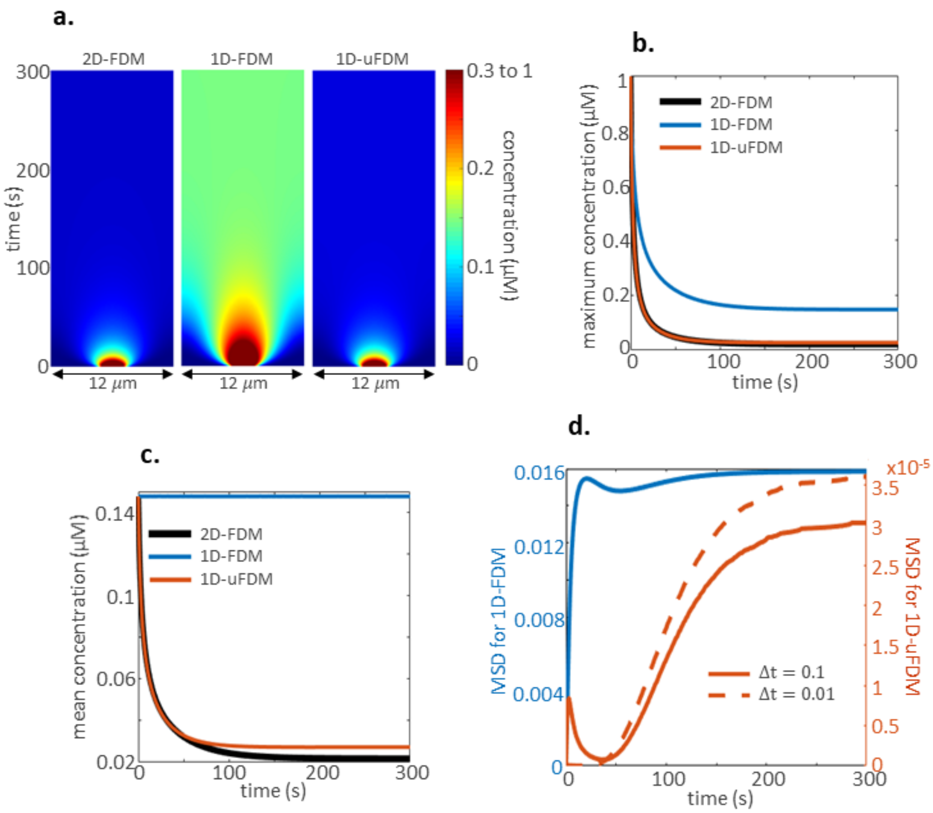

Supplementary Figure 7: Accuracy dynamics of the implicit 1D-FDM and semi-implicit 1D-uFDMs when simulating diffusion in reduced dimension models. (a) Kymographs of molecule $\boldsymbol{u}$ on the focal plane of the 2D model and in the 1D models. **(b)** Maximum concentration dynamics. **(c)** Mean concentration in the 1D solutions compared with the mean concentration in the focal plane of the 2D solution. **(d)** MSD between the focal plane in the 2D solution and the 1D solutions. 1D-FDM comparison shown in blue, 1D-uFDM comparison shown in red.

Steady state accuracy of 1D-uFDMs.

The accuracy of the 1D-uFDM solutions was investigated numerically by comparing it with the central slice of the 2D Fourier solution (See 2D Fourier solution below). The comparisons were made using three sets of *in-silico* experiments. In set 1, we asked how 1D-uFDM accuracy was affected by mesh size by fixing Δt and varying $\Delta x, \Delta y$. In set 2 we asked how accuracy was affected by increasing Δt. Here, we fixed $\Delta x, \Delta y$ and varied Δt. Finally, in set 3, we looked at the interdependence of $\Delta x, \Delta y$ and Δt by varying all three.

First we looked at the accuracy of the 1D-uFDMs when predicting steady state on the central slice of the 2D Fourier solution. Simulation set 1: To ascertain how the accuracy of the 1D-uFDMs was affected by mesh size, for each $\Delta x = 0.01, 0.02, \dots, 0.1\,\mu m$, a simulation was run for $\Delta y = 0.01, 0.02, \dots, 0.1\ \mu m$. To ensure numerical stability for all $\Delta x, \Delta y$, and for both explicit and semi-implicit numerical methods, Δt was set using the explicit 1D-uFDM stability condition (Supplementary Figure 3), $\Delta t_{-}(\Delta x, \Delta y, D, \beta) = \Delta t_{-}(0.01, 0.01, 0.1, 0.1) = \frac{1-\beta}{\mathrm{D}}\left(\frac{\Delta x^2 \Delta y^2}{2\Delta y^2 + \Delta x^2}\right) = 0.0003\ s$. The initial conditions were, $u_{1D}(x,t) = u_{1D}(x,0) = e^{-x^2}$, $u_{2D}(x,y,t) = u_{2D}(x,y,0) = e^{-(x^2+y^2)}$ (Supplementary Figures 1a and b, $\alpha = 1$). The protocol was thus, for each Δx, Δy combination: The explicit and semi-implicit 1D-uFDMs were run to $300\ s$. The 2D Fourier solution was calculated at 0 and $300\ s$. The mean squared distance (MSD) was calculated between each point on the 1D mesh and the corresponding point on the central row of the 2D mesh, for both 0 and $300\ s$. If the MSD was zero at $0\ s$ then the MSD at $300\ s$ (steady state) was recorded. Changing mesh size did not dramatically affect the accuracy of the unrestricted finite difference methods, Supplementary Figure 4a. However, increasing Δx resulted in a small increase in accuracy and increasing Δy resulted in a small decrease. For $\Delta t_{-} = 0.0003\ s$ the semi-implicit unrestricted movement finite difference method was marginally more accurate than the explicit method.

Simulation set 2: Next, we investigated the effect of Δt on the accuracy of the unrestricted movement finite difference methods by fixing $\Delta x = \Delta y = 0.1\ \mu m$ and varying Δt. $\Delta x = \Delta y = 0.1\ \mu m$ was chosen to give the largest numerically stable range of Δt values . For both semi-implicit and explicit 1D-uFDMs $\Delta t = 0.0003$ to 0.03 at intervals of 0.0027 s. As the semi-implicit 1D-uFDM is numerically stable for greater values of Δt (Supplementary Figure 3b) the semi-implicit 1D-uFDM was also run for $\Delta t = 0.04, 0.05, \dots 0.1$ s (Supplementary Figure 4b). For the explicit 1D-uFDM increasing Δt had no significant effect on the accuracy. The semi-implicit 1D-uFDM's accuracy was significantly increased with increasing Δt.

Given the difference in accuracy between the accuracy of semi-implicit and explicit 1D-FDMs for increasing Δt one would expect that fixing $\Delta t > 0.0003$ s and performing simulation set 1 again, would result in greater separation between semi-implicit and explicit 1D-uFDM accuracies than that shown in Supplementary Figure 4a. Simulations were run for $\Delta x \geq 0.05\ \mu m, \Delta y \geq 0.05\ \mu m$ and $\Delta t_{-}(0.5, 0.5, 0.1, 0.1) = \frac{1-\beta}{\mathrm{D}}\left(\frac{\Delta x^2 \Delta y^2}{2\Delta y^2 + \Delta x^2}\right) = 0.0075\ s$. The results of these simulations confirmed expectations (Supplementary Figure 4c). Fixing $\Delta x = \Delta y = 0.05\ \mu m$ and varying Δt gave further confirmation of the accuracy trends reported in Supplementary Figure 4d.

For the explicit 1D-uFDM the choice of Δx, Δy and Δt has little effect on the accuracy of the numerical method, as long as the choice satisfices the explicit 1D-uFDM stability condition. For the semi-implicit 1D-uFDM increasing Δt benefits accuracy, however to enable larger Δt one must increase Δy to ensure numerical stability. Simulation set 3 explores the interdependence of $\Delta x, \Delta y$ and Δt with regards to steady state accuracy. For these simulations, all three parameters were changed. $\Delta x = 0.01, 0.02, \dots, 0.1\ \mu m$, $\Delta y = 0.01, 0.02, \dots, 0.1\ \mu m$, and Δt was set for each Δx, Δy combination using the equation $\Delta t_{-}(\Delta x, \Delta y, D, \beta) = \Delta t_{-}(\Delta x, \Delta y, 0.1, 0.1) = \frac{1-\beta}{\mathrm{D}}\left(\frac{\Delta x^2 \Delta y^2}{2\Delta y^2 + \Delta x^2}\right)\ s$ for explicit 1D-uFDM simulations and $\Delta t_{-}(\Delta y, D, \beta) = \Delta t_{-}(\Delta y, 0.1, 0.1) = \frac{1-\beta}{\mathrm{D}} \Delta y^2$ for semi-implicit. As expected, the explicit 1D-uFDM had the same accuracy with the larger values of Δt as it did with $\Delta t = 0.0003\ \mathrm{s}$ (compare solid lines on Supplementary Figures 4a and 4e). As the stability boundary of the semi-implicit 1D-uFDM depends on Δy only, increasing Δy increases the value $\Delta t_{-}(\Delta y, 0.1, 0.1)$ and thus increases the accuracy of the semi-implicit numerical method (Supplementary Figure 4e). Continually increasing the value of Δy, and thus Δt, should eventually reduce the accuracy of the numerical method. To investigate this Δx was set to $0.1\ \mu\mathrm{m}$, Δy was increased from 0.01 to $0.5\ \mu m$, and $\Delta t_{-}(\Delta y, 0.1, 0.1) = \frac{1-\beta}{\mathrm{D}} \Delta y^2\ s$. Indeed, the minimum MSD was found to be $3.2\mathrm{x}10^{-9}$ for $\Delta y = 0.38\ \mu m$, $\Delta t = 1.2996\ s$ (Supplementary Figure 4f).

Taken together these investigations show that to improve the accuracy of the steady state estimation when using the explicit 1D-uFDM one should choose a larger Δx and smaller Δy, then set Δt with the formula $\Delta t_{-}(\Delta x, \Delta y, D, \beta) = \frac{1-\beta}{\mathrm{D}}\left(\frac{\Delta x^2 \Delta y^2}{2\Delta y^2+\Delta x^2}\right)$. For the semi-implicit method one should choose a larger Δy and set Δt using the formula $\Delta t_{-}(\Delta y, D, \beta) = \frac{1-\beta}{\mathrm{D}}\Delta y^2$.

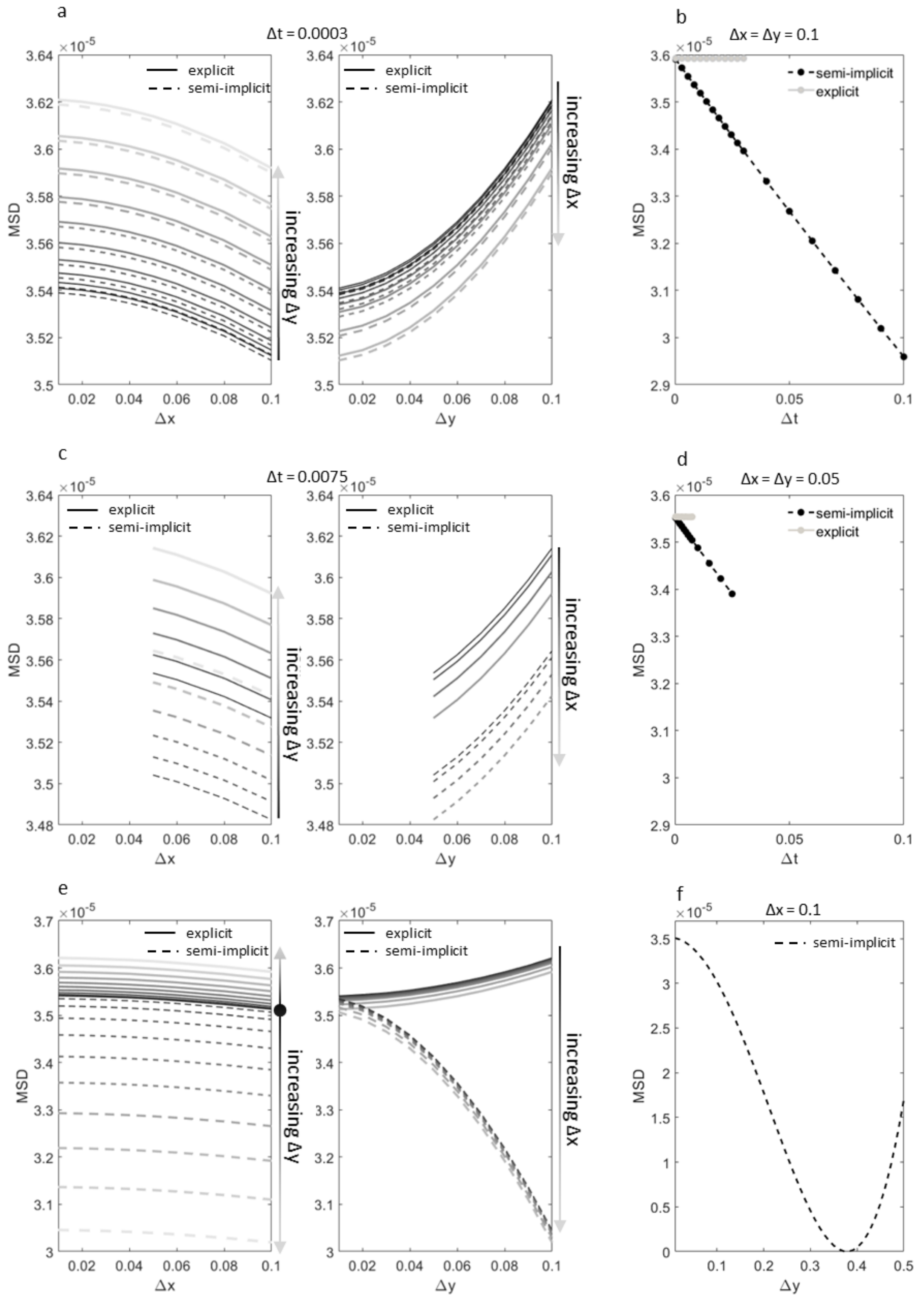

Supplementary Figure 4: Accuracy of unrestricted movement finite difference methods at steady state. Solid lines show the results of the explicit method, dotted the semi-implicit. In (a), (c) and (e) Lighter grey indicates increasing $\Delta\boldsymbol{x}$ or $\Delta\boldsymbol{y}$. **(a)** MSD between the explicit and semi-implicit 1D-FDMs and the 2D Fourier solutions at steady state, for $\Delta\boldsymbol{x} = \mathbf{0.01}, \mathbf{0.02}, \dots, \mathbf{0.1}\ \boldsymbol{\mu m}$, $\Delta\boldsymbol{y} = \mathbf{0.01}, \mathbf{0.02}, \dots, \mathbf{0.1}\ \boldsymbol{\mu m}$ and $\boldsymbol{\Delta t} = \mathbf{0.0003}$. **(b)** MSD at steady state for $\Delta\boldsymbol{x} = \boldsymbol{\Delta y} = \mathbf{0.1}\ \boldsymbol{\mu m}$, varying $\Delta\boldsymbol{t}$. **(c)** MSD between the explicit and semi-implicit 1D-FDMs and the 2D Fourier solutions at steady state, for $\Delta\boldsymbol{x} \geq \mathbf{0.05}, \boldsymbol{\Delta y} \geq \mathbf{0.05}$ and $\Delta\boldsymbol{t} = \mathbf{0.0075}$. **(d)** MSD at steady state for $\Delta\boldsymbol{x} = \boldsymbol{\Delta y} = \mathbf{0.5}\ \boldsymbol{\mu m}$, varying $\Delta\boldsymbol{t}$. **(e)** MSD at steady state for $\Delta\boldsymbol{x} = \mathbf{0.01}, \mathbf{0.02}, \dots, \mathbf{0.1}\ \boldsymbol{\mu m}$, $\Delta\boldsymbol{y} = \mathbf{0.01}, \mathbf{0.02}, \dots, \mathbf{0.1}\ \boldsymbol{\mu m}$, and $\Delta\boldsymbol{t}_{-} = \frac{\mathbf{1}-\boldsymbol{\beta}}{\mathbf{D}}\left(\frac{\Delta x^2 \Delta y^2}{2\Delta y^2 + \Delta x^2}\right)\ \boldsymbol{s}$ for explicit simulations and $\Delta\boldsymbol{t}_{-} = \frac{\mathbf{1}-\boldsymbol{\beta}}{\mathbf{D}}\Delta\boldsymbol{y}^{\mathbf{2}}\ \boldsymbol{s}$ for semi-implicit. **(f)** MSD at steady state for semi-implicit 1D-uFDM, $\Delta\boldsymbol{x} = \mathbf{0.1}\ \boldsymbol{\mu m}$, $\Delta\boldsymbol{y} = \mathbf{0.01}, \mathbf{0.02}, \dots \mathbf{0.5}\ \boldsymbol{\mu m}$, and $\Delta\boldsymbol{t}_{-}(\boldsymbol{\Delta y}, \mathbf{0.1}, \mathbf{0.1}) = \frac{\mathbf{1}-\boldsymbol{\beta}}{\mathbf{D}}\Delta\boldsymbol{y}^{\mathbf{2}}\ \boldsymbol{s}$.

2D Fourier solution.

Consider a 2D space $x \in [-L, L]$ and $y \in [-L, L]$ with periodic boundary conditions. The diffusion equation in 2D space is $\frac{\partial}{\partial t}v(x,y,t) = D\left(\frac{\partial^2}{\partial x^2}v(x,y,t) + \frac{\partial^2}{\partial y^2}v(x,y,t)\right)$, with periodic boundary conditions,

$$
\begin{aligned}
&v(-L,y,t) = v(L,y,t)\\
&v(x,-L,t) = v(x,L,t)\\
&\frac{\partial}{\partial x}v(x,y,t)\Big|_{-L} = \frac{\partial}{\partial x}v(x,y,t)\Big|_{L}\\
&\frac{\partial}{\partial y}v(x,y,t)\Big|_{-L} = \frac{\partial}{\partial y}v(x,y,t)\Big|_{L}
\end{aligned}
$$

Separation of variables states $v(x,y,t)$ will have the form, $v(x,y,t) = X(x)Y(y)T(t)$. Substitute $v(x,y,t) = X(x)Y(y)T(t)$ into the 2D diffusion equation to get, $\frac{1}{D\,T(t)}\frac{\partial}{\partial t}T(t) = \frac{1}{X(x)}\frac{\partial^2}{\partial x^2}X(x) + \frac{1}{Y(y)}\frac{\partial^2}{\partial y^2}Y(y) = -\lambda = -(\lambda_n + \lambda_m)$. $T(t)$ and $X(x)$ have the same solution as they did when considering the 1D diffusion equation, and the solution for $Y(y)$ will have the same form as $X(x)$. However, for the 2D case $\lambda_n = \left(\frac{n\pi}{L}\right)^2$, $\lambda_m = \left(\frac{m\pi}{L}\right)^2$ and $\lambda = \left(\frac{n\pi}{L}\right)^2 + \left(\frac{m\pi}{L}\right)^2$.

$$
\begin{aligned}
&T(t) = Ce^{-\left(\left(\frac{n\pi}{L}\right)^2 + \left(\frac{m\pi}{L}\right)^2\right)Dt}\\
&X(x) = k_2\\
&X_n(x) = k_{1n}\cos\left(\frac{n\pi}{L}x\right) + k_{2n}\sin\left(\frac{n\pi}{L}x\right)\\
&Y(y) = k_4\\
&Y_n(y) = k_{1m}\cos\left(\frac{m\pi}{L}y\right) + k_{2m}\sin\left(\frac{m\pi}{L}y\right)
\end{aligned}
$$

The corresponding solutions for $v(x,y,t)$ are $v_{nm}(x,y,t)$,

$$
\begin{aligned}
&v_{00}(x,y,t) = C_{00}\\
&v_{0m}(x,y,t) = C_{0m}\left(A_m\cos\left(\frac{m\pi}{L}y\right) + B_m\sin\left(\frac{m\pi}{L}y\right)\right)e^{-\left(\frac{m\pi}{L}\right)^2 Dt}\\
&v_{n0}(x,y,t) = C_{n0}\left(A_n\cos\left(\frac{n\pi}{L}x\right) + B_n\sin\left(\frac{n\pi}{L}x\right)\right)e^{-\left(\frac{n\pi}{L}\right)^2 Dt}\\
&v_{nm}(x,y,t) = \left(A_n\cos\left(\frac{n\pi}{L}x\right) + B_n\sin\left(\frac{n\pi}{L}x\right)\right)\left(A_m\cos\left(\frac{m\pi}{L}y\right) +\right.\\
&\qquad\qquad\qquad \left.B_m\sin\left(\frac{m\pi}{L}y\right)\right)e^{-\left(\left(\frac{n\pi}{L}\right)^2 + \left(\frac{m\pi}{L}\right)^2\right)Dt}
\end{aligned}
$$

Using the principle of superposition, we write the general solution to the 2D diffusion equation with periodic boundary conditions,

$$v(x,y,t) = v_{00}(x,y,t) + \sum_{m=1,2\ldots} v_{0m}(x,y,t) + \sum_{n=1,2\ldots} v_{n0}(x,y,t) + \sum_{n,m=1,2\ldots} v_{nm}(x,y,t)$$

Again, we use Fourier analysis find the Fourier coefficients and solve the general solution for a given initial condition. The general solution for the 2D diffusion equation with initial condition $v(x,y,0) = e^{-(x^2+y^2)}$ is,

$$v(x,y,t) = C_{00} + \sum_{m=1,2\ldots} A_{0m} \cos\left(\frac{m\pi}{L}y\right) e^{-\left(\frac{m\pi}{L}\right)^2 Dt} + \sum_{n=1,2\ldots} A_{n0} \cos\left(\frac{n\pi}{L}x\right) e^{-\left(\frac{n\pi}{L}\right)^2 Dt} + \sum_{n,m=1,2\ldots} A_{nm} \cos\left(\frac{n\pi}{L}x\right) \cos\left(\frac{m\pi}{L}y\right) e^{-\left(\left(\frac{n\pi}{L}\right)^2 + \left(\frac{m\pi}{L}\right)^2\right)Dt}$$

$$C_{00} = \frac{\pi}{(2L)^2} \operatorname{erf}(L)^2$$

$$A_{0m} = \frac{-\pi}{i(2L)^2} \operatorname{erf}(L)\, e^{-\left(\frac{m\pi}{2L}\right)^2} \left(\operatorname{erfi}\left(\frac{L}{i} - \frac{m\pi}{2L}\right) + \operatorname{erfi}\left(\frac{L}{i} + \frac{m\pi}{2L}\right)\right)$$

$$A_{n0} = \frac{-\pi}{i(2L)^2} \operatorname{erf}(L)\, e^{-\left(\frac{n\pi}{2L}\right)^2} \left(\operatorname{erfi}\left(\frac{L}{i} - \frac{n\pi}{2L}\right) + \operatorname{erfi}\left(\frac{L}{i} + \frac{n\pi}{2L}\right)\right)$$

$$A_{nm} = \frac{-\pi}{(2L)^2} e^{-\left(\frac{n\pi}{2L}\right)^2 - \left(\frac{m\pi}{2L}\right)^2} \left(\operatorname{erfi}\left(\frac{L}{i} - \frac{n\pi}{2L}\right) + \operatorname{erfi}\left(\frac{L}{i} + \frac{n\pi}{2L}\right)\right)\left(\operatorname{erfi}\left(\frac{L}{i} - \frac{m\pi}{2L}\right) + \operatorname{erfi}\left(\frac{L}{i} + \frac{m\pi}{2L}\right)\right)$$

Accuracy dynamics of 1D-uFDMs.

Next, we compare the dynamics of the 1D-uFDMs to see how faithfully they estimate the approach to steady state. Similar to the steady state accuracy investigation we performed three sets of simulations. Simulation set 1: Investigate the time course dependence of 1D-uFDMs accuracy, and its relationship to mesh size. As calculating the Fourier solution every second for $300\,s$ is computationally time consuming we chose to investigate fewer mesh sizes than in the steady state accuracy investigation. For each $\Delta x = 0.02, 0.04, \ldots, 0.1\,\mu m$, a simulation was run for $\Delta y = 0.02, 0.04, \ldots, 0.1\,\mu m$. Again, to ensure numerical stability for all $\Delta x, \Delta y$, and for both explicit and semi-implicit numerical methods, Δt was set using the explicit 1D-uFDM stability condition (Supplementary Figure 3). $\Delta t\_(\Delta x, \Delta y, D, \beta) = \Delta t\_(0.01, 0.01, 0.1, 0.1) = 0.0003\,s$. The initial conditions were $u_{1D}(x,t) = u_{1D}(x,0) = e^{-x^2}$, $u(x,y,t) = u(x,y,0) = e^{-(x^2+y^2)}$ (Supplementary Figures 1a and b, $\alpha = 1$). The protocol was thus, for each Δx, Δy combination: The explicit and semi-implicit 1D-uFDMs were run to $300\,s$. As Δt did not divide exactly into one, the 1D-uFDMs concentration profiles were recorded at close to one second intervals, and the times of data collection were recorded. The 2D Fourier solutions were calculated at the same times as the 1D-uFDMs' data was collected. For every time point the MSD was calculated between the 1D-uFDMs and the central row of the 2D Fourier solution.

Supplementary Figure 5a shows the MSD time evolution. As with the steady state MSD analysis, MSD dynamics were similar for all Δx, Δy tested, for both the explicit and semi-implicit 1D-uFDMs. In all cases the MSD increased rapidly at around $30\,s$. To understand the cause of the rapid MSD increase we calculated the absolute distance between each point of the semi-implicit 1D-uFDM and the central row of the 2D Fourier solution, at every second, for $\Delta x = 0.1\,\mu m$, $\Delta y = 0.02\,\mu m$ as this had the smallest MSD at steady state (Supplementary Figures 4a and 5c). The absolute distance analysis showed that the absolute distance began to increase at the boundary edges and propagated through the mesh. Looking at the semi-implicit 1D-uFDM concentration profile we see that the increase in absolute distance at the boundaries corresponds to the rate of change of concentration at the boundaries (compare Supplementary Figures 5c and 5d, see also Supplementary Figure 5e). This is unsurprising as estimating concentrations $u^{\tau}_{\sqrt{(N/2\,\Delta x)^2+\Delta y^2}}$, which would lie at the boundaries of the 2D domain (black circle Supplementary Figure 5b), involves a 1D interpolation into space without any concentration information (orange circle Supplementary Figure 5b). As the curvature at the boundary increases so too does the interpolation error.

Looking more closely at the MSD time course data (Supplementary Figure 5a), we see a small peak before the edge error is detected (Supplementary Figures 6a and 6b). This initial MSD peak has a strong Δy dependency. To ask if this initial MSD peak was a result of interpolation error we looked at the interpolation accuracy data (Supplementary Figure 2, row $\alpha = 1$, $\Delta x = 0.1\ \mu m$). This data showed that the interpolation accuracy does decrease with increasing Δy, and that the decrease is predominantly generated in the central region, around the location of the peak. However the size of the initial MSD peak is not accounted for by the interpolation inaccuracy on the initial concentration (Supplementary Figure 5a, row 2, column 1, $\alpha = 1$, $\Delta x = 0.1\ \mu m$, $\mathrm{L} = 12\ \mu m$, divide max SSD in figure by $L/\Delta x = 120$ to get a maximum MSD around 10^{-13}).
The 1D-uFDMs are composed of two parts, the 1D-FDM (highlighted in blue text in the equations below), and the terms utilising the interpolated concentrations.

$$u_n^{\tau+1} = u_n^\tau + d_x(u_{n-1}^\tau - 2u_n^\tau + u_{n+1}^\tau) + 2d_y\left(u^\tau_{\sqrt{(n\Delta x)^2+\Delta y^2}} - u_n^\tau\right)$$
$$u_n^{\tau+1} = u_n^\tau + d_x(u_{n-1}^{\tau+1} - 2u_n^{\tau+1} + u_{n+1}^{\tau+1}) + 2d_y\left(u^\tau_{\sqrt{(n\Delta x)^2+\Delta y^2}} - u_n^\tau\right)$$

To ask if the error inherent in the 1D-FDMs could account for the initial MSD peak the 1D-uFDMs, we compared the solutions of the 1D-FDMs with the 1D Fourier solutions for the diffusion equation (1D Fourier solution derived in 1D Fourier solution below). To enable direct error comparisons between the 1D-FDMs inherent error analysis and the 1D-uFDMs error analysis the 1D-FDMs were run for $300\ s$, with $\Delta x = 0.02, 0.04, \ldots, 0.1\ \mu m$, $\Delta t = 0.0003\ s$ and initial condition $u_{1D}(x,t) = u_{1D}(x,0) = e^{-x^2}$. Again, as Δt does not divide exactly into one, the 1D-FDMs concentration profiles were recorded at close to one second intervals, and the times of data collection were recorded. The 1D Fourier solutions were calculated at the same times. For every time point, the MSDs and SDs were calculated between the 1D-FDMs and the 1D Fourier solutions. Supplementary Figure 6c shows the 1D-FDMs MSD has initial MSD peaks comparable to the 1D-uFDMs initial MSD peaks for $\Delta y = 0.02, 0.04\ \mu m$, with the initial MSD peaks increasing in size with Δx (compare Supplementary Figures 6a, 6b and 6c). As Δy increases further interpolation inaccuracies are combined with the 1D-FDMs inherent error to increase the initial 1D-uFDMs MSD peaks, nonetheless these increased peak sizes remain within the same order of magnitude as the 1D-FDMs MSD peaks. To further confirm the hypothesis that the 1D-uFDMs initial MSD peaks could be attributed to the 1D-FDMs inherent error we compared 1D-FDMs and 1D-uFDMs SD kymographs of the first 20 s of SD data (Supplementary Figure 6d). Indeed, the 1D-uFDMs SD kymographs showed close resemblance to the 1D-uFDM SD kymographs for smaller Δy, with the largest SDs at the points of greatest curvature in the concentration profile. The last point of note is that the 1D-FDMs MSDs do not increase again after the initial peak (Supplementary Figure 6c) confirming that the dominant error increase in 1D-uFDMs MSD is due to interpolation inaccuracy at the domain edges (Supplementary Figure 5).

Simulation set 2: In the steady state analysis it was found that increasing Δt improved the accuracy of the semi-implicit 1D-uFDM when estimating the homogeneous steady state, but not the explicit 1D-uFDM (Supplementary Figure 4b). To ask if the error inherent in the 1D-FDMs can account for the differences between the explicit and semi-implicit 1D-uFDM MSDs at steady state we performed a time course MSD analysis on 1D-FDMs and 1D-uFDMs with fixed $\Delta x = \Delta y = 0.1\ \mu m$ and increased Δt from $0.0003\ s$ (the Δt value in simulation set 1) to $0.03\ \mathrm{s}$ for explicit 1D-FDM and 1D-uFDM and to $0.1\ \mathrm{s}$ for the implicit 1D-FDM and semi-implicit 1D-uFDM, ensuring a range of values for Δt while retaining numerical stability. We found that the error inherent in 1D-FDMs followed the same trend as did the steady state 1D-uFDMs MSD, namely increasing Δt in the explicit 1D-FDM had no effect on the MSD, and increasing Δt in the implicit 1D-FDM had a dramatic effect on the MSD (compare Supplementary Figures 4b and 6e). The 1D-FDM and 1D-uFDM initial MSD peaks followed the same trend, as expected (compare Supplementary Figures 6e and 6f inset). Looking at the entire MSD time course for the semi-implicit 1D-uFDM (Supplementary Figure 6f) we see that, for the values of Δt chosen, the increase in MSD initial peak offsets the steady state MSD.

Simulation set 3: For completion we analysed the MSD dynamics in the semi-implicit 1D-uFDM for fixed $\Delta x = 0.1\ \mu m$, increasing $\Delta y = 0.01, 0.02, \ldots 0.5\ \mu m$, and increasing $\Delta t\_(\Delta y, D, \beta) = \Delta t\_(\Delta y, 0.1, 0.1) = \frac{1-\beta}{\mathrm{D}} \Delta y^2\ s$ (steady state MSD shown in Supplementary Figure 4f). Increasing Δy (and Δt) resulted in an increasing MSD initial peak, it did not have a minimum similar to the MSD at steady state, indicating the optimal Δy for minimum MSD at steady state is found at a balance point between 1D-FDM error and interpolation boundary error (Supplementary Figure 6g).

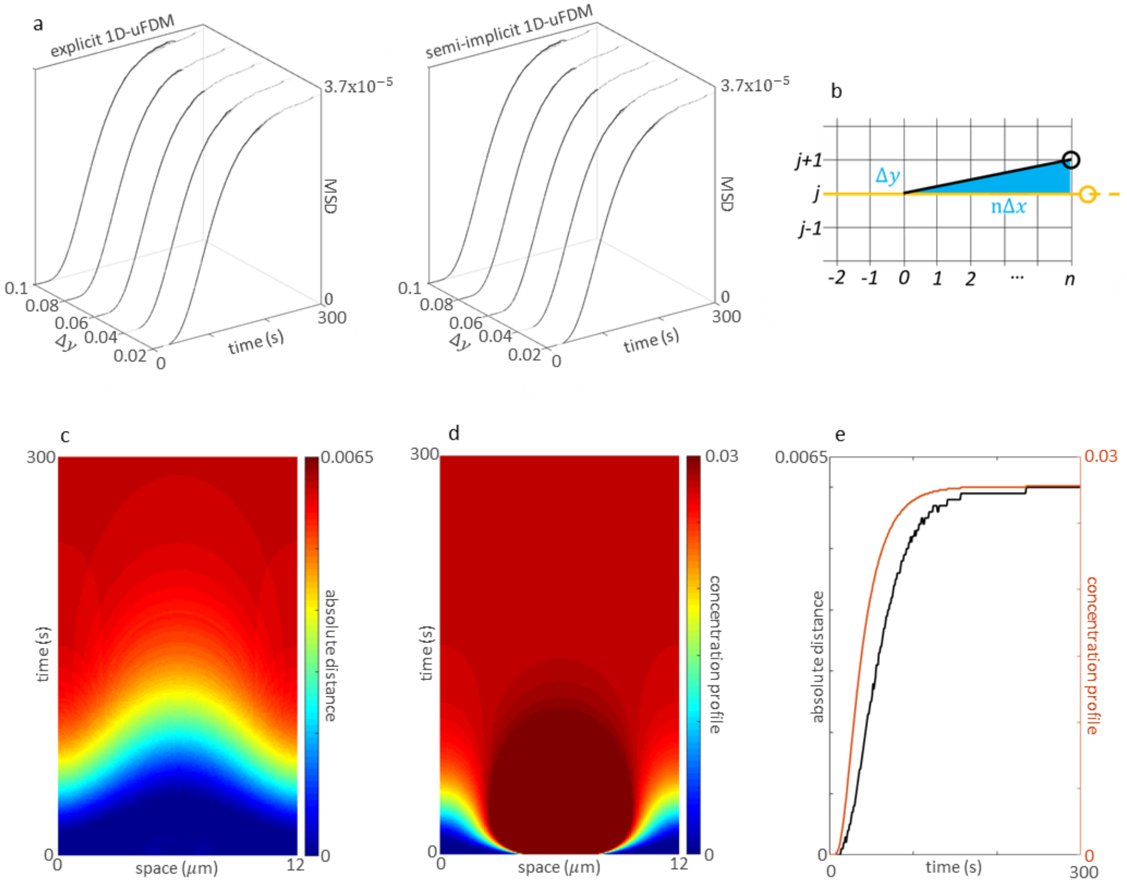

Supplementary Figure 5: Accuracy dynamics of unrestricted movement finite difference methods: Interpolation error. (a) MSD between the explicit and semi-implicit 1D-FDMs and the 2D Fourier solutions for $\boldsymbol{\Delta x = 0.02, 0.04, \ldots, 0.1\ \mu m}$, $\boldsymbol{\Delta y = 0.02, 0.04, \ldots, 0.1\ \ \mu m}$ and $\boldsymbol{\Delta t = 0.0003\ s}$. For each $\boldsymbol{\Delta y}$ the five MSD curves for $\Delta \boldsymbol{x} = \boldsymbol{0.02, 0.04, \ldots, 0.1\ \mu m}$ are drawn on top of each other. **(b)** Cartoon showing the estimation of $\boldsymbol{u^{\tau}_{\sqrt{(N/2\ \Delta x)^2 + \Delta y^2}}}$ at the boundary. **(c)** Kymograph of the absolute distance between each point of the semi-implicit 1D-uFDM and the central row of the 2D Fourier solution, at every second, for $\boldsymbol{\Delta x = 0.1\ \mu m}$, $\boldsymbol{\Delta y = 0.02\ \mu m}$, $\boldsymbol{\Delta t = 0.0003\ s}$. (d) Kymograph, with upper threshold defined by the steady state concentration, of the concentration profile of the semi-implicit 1D-uFDM for $\boldsymbol{\Delta x = 0.1\ \mu m}$, $\boldsymbol{\Delta y = 0.02\ \mu m}$, $\boldsymbol{\Delta t = 0.0003\ s}$. **(e)** Graph of the absolute distance and concentration profile at the boundary.

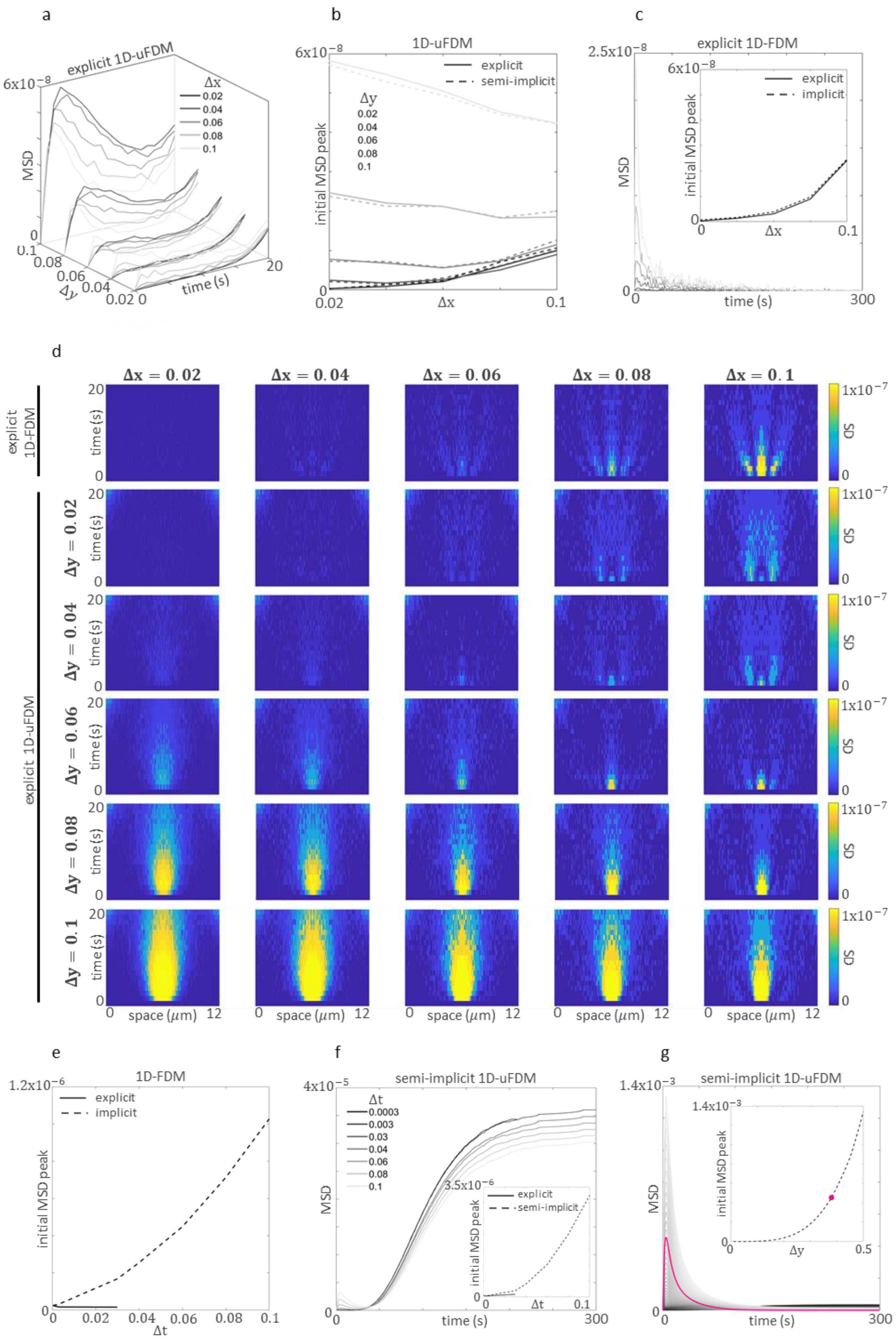
a
explicit 1D-uFDM
6x10⁻⁸
MSD
Δx
0.02
0.04
0.06
0.08
0.1
Δy
time (s)
20
b
1D-uFDM
explicit
semi-implicit
initial MSD peak
Δy
Δx
c
explicit 1D-FDM
2.5x10⁻⁸
explicit
implicit
time (s)
300
d
Δx = 0.02
Δx = 0.04
Δx = 0.06
Δx = 0.08
Δx = 0.1
explicit 1D-FDM
explicit 1D-uFDM
Δy = 0.02
Δy = 0.04
Δy = 0.06
Δy = 0.08
Δy = 0.1
time (s)
space (μm)
SD
1x10⁻⁷
e
1D-FDM
1.2x10⁻⁶
explicit
implicit
initial MSD peak
Δt
f
semi-implicit 1D-uFDM
4x10⁻⁵
Δt
0.0003
0.003
0.03
0.04
0.06
0.08
0.1
MSD
3.5x10⁻⁶
explicit
semi-implicit
initial MSD peak
time (s)
g
semi-implicit 1D-uFDM
1.4x10⁻³
MSD
initial MSD peak
Δy
0.5
time (s)
300

Supplementary Figure 6: Accuracy dynamics of unrestricted movement finite difference methods: 1D-FDM error. (a) Initial MSD peaks between the explicit 1D-uFDM and the 2D Fourier solutions, for $\Delta x = \mathbf{0.02, 0.04, \dots, 0.1}\ \boldsymbol{\mu m}$, $\Delta y = \mathbf{0.02, 0.04, \dots, 0.1}\ \boldsymbol{\mu m}$ and $\boldsymbol{\Delta t} = \mathbf{0.0003}\ \boldsymbol{s}$. **(b)** Values of initial MSD peaks for explicit and semi-implicit 1D-uFDM, for $\Delta \boldsymbol{x} = \mathbf{0.02, 0.04, \dots, 0.1}\ \boldsymbol{\mu m}$, $\Delta \boldsymbol{y} = \mathbf{0.02, 0.04, \dots, 0.1}\ \boldsymbol{\mu m}$ and $\boldsymbol{\Delta t} = \mathbf{0.0003}\ \boldsymbol{s}$. **(c)** MSD between the explicit 1D-FDM and the 1D Fourier solutions, for $\Delta \boldsymbol{x} = \mathbf{0.02, 0.04, \dots, 0.1}\ \boldsymbol{\mu m}$ and $\boldsymbol{\Delta t} = \mathbf{0.0003}\ \boldsymbol{s}$. Lighter grey indicates larger $\boldsymbol{\Delta x}$, key in Supplementary Figure 6a. **(c inset)** Values of initial MSD peaks for explicit and semi-implicit 1D-FDM. **(d)** SD kymographs showing the initial SD peak for $\Delta \boldsymbol{x} = \mathbf{0.02, 0.04, \dots, 0.1}\ \boldsymbol{\mu m}$, $\boldsymbol{\Delta t} = \mathbf{0.0003}\ \boldsymbol{s}$. Row 1 explicit 1D-FDM, rows 2 to 6 explicit 1D-uFDM for $\Delta \boldsymbol{y} = \mathbf{0.02, 0.04, \dots, 0.1}\ \boldsymbol{\mu m}$. **(e)** Initial MSD peaks for 1D-FDM, $\Delta \boldsymbol{x} = \boldsymbol{\Delta y} = \mathbf{0.1}\ \boldsymbol{\mu m}$, varying $\Delta \boldsymbol{t}$. **(f)** MSD for semi-implicit 1D-uFDM, $\Delta \boldsymbol{x} = \boldsymbol{\Delta y} = \mathbf{0.1}\ \boldsymbol{\mu m}$, varying $\Delta \boldsymbol{t}$. **(f inset)** Initial MSD peaks for 1D-uFDM, $\Delta \boldsymbol{x} = \boldsymbol{\Delta y} = \mathbf{0.1}\ \boldsymbol{\mu m}$, varying $\Delta \boldsymbol{t}$. **(g)** MSD for semi-implicit 1D-uFDM, $\Delta \boldsymbol{x} = \mathbf{0.1}\ \boldsymbol{\mu m}$, $\Delta \boldsymbol{y} = \mathbf{0.01, 0.02, \dots 0.5}\ \boldsymbol{\mu m}$, and $\Delta \boldsymbol{t}_{-}(\boldsymbol{\Delta y}, \mathbf{0.1}, \mathbf{0.1}) = \frac{\mathbf{1}-\boldsymbol{\beta}}{\mathbf{D}} \Delta \boldsymbol{y}^{\mathbf{2}}\ \boldsymbol{s}$. **(g inset)** Initial MSD peaks for semi-implicit 1D-uFDM, $\Delta \boldsymbol{x} = \mathbf{0.1}\ \boldsymbol{\mu m}$, $\Delta \boldsymbol{y} = \mathbf{0.01, 0.02, \dots 0.5}\ \boldsymbol{\mu m}$, and $\Delta \boldsymbol{t}_{-}(\boldsymbol{\Delta y}, \mathbf{0.1}, \mathbf{0.1}) = \frac{\mathbf{1}-\boldsymbol{\beta}}{\mathbf{D}} \Delta \boldsymbol{y}^{\mathbf{2}}\ \boldsymbol{s}$.

1D Fourier solution.

Regarding notation: For the finite difference scheme, u_n^τ denotes the concentration of a species on mesh point n at time τ. To avoid confusion, here we define the notation for the Fourier solution. Let $v(x,t)$ or $v(x,y,t)$ describe the concentration of a species at position x or (x,y) at time t. The Fourier general solution will be comprised of the sum of fundamental solutions, using the principle of superposition. $v_n(x,t)$ or $v_n(x,y,t)$ will denote the nth fundamental solution. For this investigation, the 1D initial condition was $v(x,0) = e^{-x^2}$, 2D $v(x,y,0) = e^{-(x^2+y^2)}$ (Supplementary Figure 1a and b, $\alpha = 1$), and $D = 0.1\ \mu m^2 s^{-1}$.

The 1D Fourier solution informs the 2D solution so we will present that first. Consider a 1D space $-L$ to L, with periodic boundary conditions. Diffusive movement of $v(x,t)$ in the 1D space is given by the equation, $\frac{\partial}{\partial t} v(x,t) = D \frac{\partial^2}{\partial x^2} v(x,t)$, where D is the diffusion coefficient. The periodic boundary conditions are,

$$v(-L,t) = v(L,t) \qquad \textit{Boundary condition 1}$$

$$\left.\frac{\partial}{\partial x} v(x,t)\right|_{-L} = \left.\frac{\partial}{\partial x} v(x,t)\right|_{L} \qquad \textit{Boundary condition 2}$$

The diffusion equation can be solved by separation of variables and Fourier series expansions [1, 2]. Separation of variables states $v(x,t)$ will have the form, $v(x,t) = X(x)T(t)$. Substitute $v(x,t) = X(x)T(t)$ into the diffusion equation to get,

$$\frac{1}{D\,T(t)} \frac{\partial}{\partial t} T(t) = \frac{1}{X(x)} \frac{\partial^2}{\partial x^2} X(x) \qquad \textit{Equation 3}$$

Both sides of the Equation 3 must equal a constant as one side depends only on time and the other depends only on space. Let the constant be $-\lambda$. The time aspect of Equation 3 is, $\frac{\partial}{\partial t} T(t) + \lambda\, D\, T(t) = 0$, which has the solution, $T(t) = Ce^{-\lambda D t}$, where C is an arbitrary constant. The space aspect of Equation 3 is,

$$\frac{\partial^2}{\partial x^2} X(x) + \lambda\, X(x) = 0 \qquad \textit{Equation 4}$$

There are three solutions to Equation 4, one for $\lambda < 0$, $\lambda = 0$ and $\lambda > 0$. The validity of these solutions are tested using boundary conditions 1 and 2. For $\lambda < 0$, $X(x) = k_1 \cosh\left(\sqrt{-\lambda}\, x\right) + k_2 \sinh\left(\sqrt{-\lambda}\, x\right)$, which has only trivial solutions. For $\lambda = 0$, $X(x) = k_1 x + k_2$. Applying Boundary Conditions 1 and 2 we get the fundamental solution,

$$v_0(x,t) = C_0 \qquad \text{Equation 5}$$

For $\lambda > 0$, Equation 4 has the solution, $X(x) = k_1 \cos(\sqrt{\lambda}\, x) + k_2 \sin(\sqrt{\lambda}\, x)$. Applying Boundary Condition 1 gives, $k_1 \cos(-\sqrt{\lambda}\, L) + k_2 \sin(-\sqrt{\lambda}\, L) = k_1 \cos(\sqrt{\lambda}\, L) + k_2 \sin(\sqrt{\lambda}\, L)$. As $\cos$ is even we get, $-k_2 \sin(\sqrt{\lambda}\, L) = k_2 \sin(\sqrt{\lambda}\, L)$. This equality holds if $k_2 = 0$, or $\lambda_n = \left(\frac{n\pi}{L}\right)^2$, $n = 1,2,3 \ldots$. The solution $X(x) = k_1 \cos(\sqrt{\lambda}\, x) + k_2 \sin(\sqrt{\lambda}\, x)$ becomes $X_n(x) = k_{1n} \cos\left(\frac{n\pi}{L} x\right) + k_{2n} \sin\left(\frac{n\pi}{L} x\right)$, which satisfies Boundary Condition 2. Note the solution for $k_2 = 0$ is accounted for in $X_n(x)$ because of the principle of superposition. Thus we have the set of fundamental solutions,

$$v_n(x,t) = \left(A_n \cos\left(\frac{n\pi}{L} x\right) + B_n \sin\left(\frac{n\pi}{L} x\right)\right) e^{-\left(\frac{n\pi}{L}\right)^2 Dt} \qquad \text{Equation 6}$$

where $A_n = k_{1n} C_n$ and $B_n = k_{2n} C_n$.
Using the principle of superposition, we write the general solution to the diffusion equation with Boundary Conditions 1 and 2 as a linear composition of all solutions (Equation 5 and Equation 6), $v(x,t) = v_0(x,t) + \sum_{n=1,2\ldots} v_n(x,t)$,

$$v(x,t) = C_0 + \sum_{n=1,2\ldots} \left[\left(A_n \cos\left(\frac{n\pi}{L} x\right) + B_n \sin\left(\frac{n\pi}{L} x\right)\right) e^{-\left(\frac{n\pi}{L}\right)^2 Dt}\right] \qquad \text{Equation 7}$$

We use the initial condition and Fourier analysis to determine the coefficients of Equation 7, and thus obtain the solution of the diffusion equation with the given initial condition. The initial condition is $(x,0) = e^{-x^2}$. At $t = 0$ Equation 7 becomes a Fourier series, $e^{-x^2} = C_0 + \sum_{n=1,2\ldots} \left[A_n \cos\left(\frac{n\pi}{L} x\right) + B_n \sin\left(\frac{n\pi}{L} x\right)\right]$. The initial condition is an even function and so $B_n = 0 \; \forall \, n$. Fourier analysis states that $C_0 = \frac{1}{2L} \int_{-L}^{L} e^{-x^2}\, dx$, giving, and $A_n = \frac{1}{L} \int_{-L}^{L} \cos\left(\frac{n\pi}{L} x\right) e^{-x^2}\, dx$. Thus, the general solution for the 1D diffusion equation with initial condition $v(x,0) = e^{-x^2}$ and periodic boundary conditions is,

$$v(x,t) = C_0 + \sum_{n=1,2\ldots} \left[A_n \cos\left(\frac{n\pi}{L} x\right) e^{-\left(\frac{n\pi}{L}\right)^2 Dt}\right]$$
$$C_0 = \frac{1}{2L} \sqrt{\pi}\, \mathrm{erf}(L)$$
$$A_n = \frac{1}{2L} i\sqrt{\pi}\, e^{-\left(\frac{n\pi}{2L}\right)^2} \left(\mathrm{erfi}\left(\frac{n\pi}{2L} - iL\right) - \mathrm{erfi}\left(\frac{n\pi}{2L} + iL\right)\right)$$

Accuracy of the 1D-FDM and 1D-uFDM when simulating FRAP.

The accuracy analysis for the 1D diffusion solutions when solving for an initial peak or an initial trough (i.e. the FRAP solution) is almost identical (compare Fig. 2C and E with Supplementary Figures 8b and c). Both the inherent 1D-FDM error and edge error (see Accuracy dynamics of 1D-uFDMs above) increase proportionally with the concentration's curvature, and in both the peak and trough profiles the curvature is described by the same equation, $e^{-(x^2+y^2)}$ in the 2D case, e^{-x^2} for 1D. Both peak and trough solutions are subject to the same diffusion coefficient so the curvature of the profiles change at the same rate giving the same accuracy analysis results.

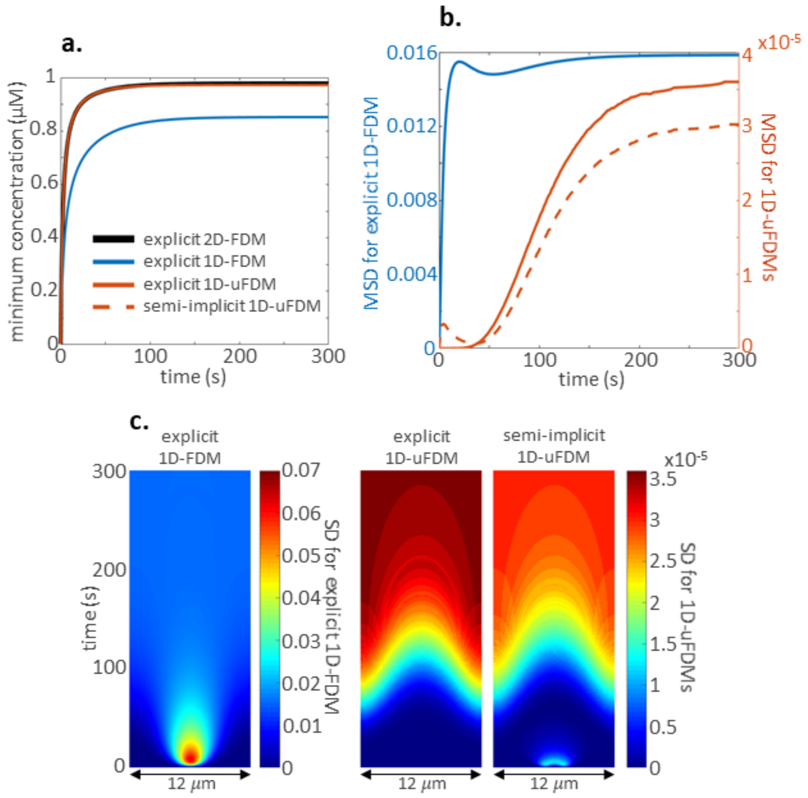

Supplementary Figure 8: Accuracy dynamics of the 1D-FDM and 1D-uFDMs when simulating FRAP in reduced dimension models. Figures support Fig. 3B-E. **(a)** Time course of the minimum concentration, i.e. the concentration at the centre of the ROI. **(b)** The MSD between the explicit 2D solution and the explicit 1D-FDM, explicit 1D-uFDM and semi-implicit 1D-uFDM. **(c)** Kymographs of the SD between the explicit 2D solution and the explicit 1D-FDM, explicit 1D-uFDM and semi-implicit 1D-uFDM.

Reaction-diffusion comparisons and the effect of geometry.

The explicit 1D-uFDM captures RD dynamics through the focal plane more accurately than the explicit 1D-FDM (Supplementary Figure 9), with an MSD an order of magnitude smaller than that of the 1D-FDM for all α (Supplementary Figures 9c and f). Analysis of implicit 1D-FDM and semi-implicit 1D-uFDM comparisons gave almost identical results to the explicit comparisons, data not shown.

Recall, if the zero flux assumption holds, then the 1D-FDM solution is an accurate approximation of molecular dynamics through the focal plane of the 2D membrane. Thus the 1D-FDM solution to the RD equations is accurate if we were investigating an initial pulse on the surface of a spherical cell, for example (Fig. 1A, E). Supplementary Figure 10 shows the RD equations, $\alpha = 3$, solved on the body of an elongated cell using the square mesh and on a spherical cell using a spherical mesh. The same RD equations produce a patch on the square mesh and a ring on the spherical mesh.

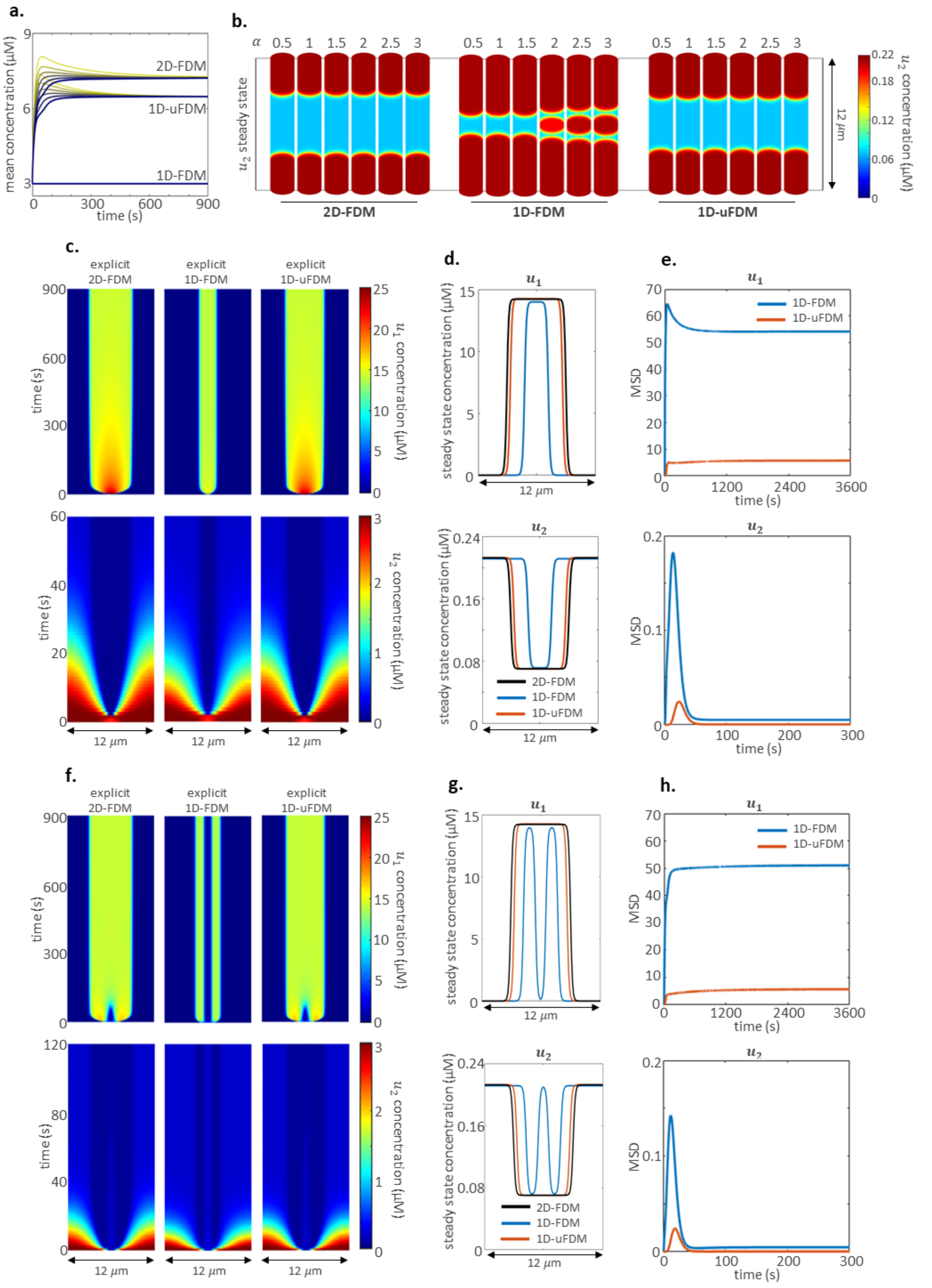

Supplementary Figure 9: Accuracy dynamics of the 1D-FDM and 1D-uFDMs when solving RD equations. Figures support Fig. 4B-D. **(a)** Mean concentration in the 1D solutions compared with the mean concentration in the focal plane of the 2D solution. **(b)** u_2 steady state colorplots for the 2D solution on the focal plane, and 1D solutions. $\alpha = 0.5$ in panels (c), (d) and (e). **(c)** Kymographs of the u_1 and u_2 concentrations in the focal plane of the explicit 2D solution and 1D

solutions. **(d)** $\boldsymbol{u_1}$ and $\boldsymbol{u_2}$ concentration profiles at steady state. **(e)** $\boldsymbol{u_1}$ and $\boldsymbol{u_2}$ MSD dynamics, comparing the concentrations in the focal plane of the 2D solution and the 1D solutions. $\boldsymbol{\alpha = 3}$ in panels (f), (g) and (h). **(f)** Kymographs of the $\boldsymbol{u_1}$ and $\boldsymbol{u_2}$ concentrations in the focal plane of the 2D solution and 1D solutions. **(g)** $\boldsymbol{u_1}$ and $\boldsymbol{u_2}$ concentration profiles at steady state. **(h)** $\boldsymbol{u_1}$ and $\boldsymbol{u_2}$ MSD dynamics, comparing the concentrations in the focal plane of the 2D solution and the 1D solutions.

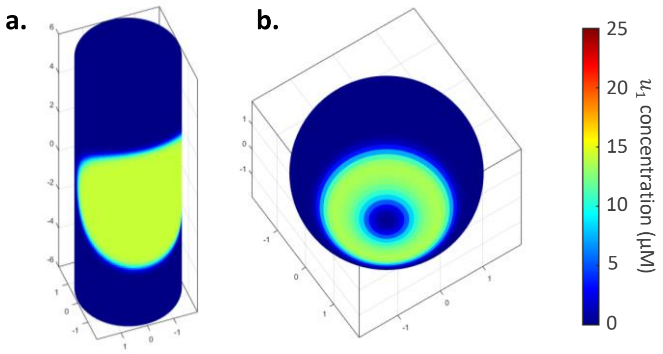

Supplementary Figure 10: The effect of geometry on the RD solutions. Compare with Fig. 4C-D, $\alpha = 3$. **(a)** Final steady state of the 2D-FDM solution to the RD equations, initial condition $\boldsymbol{\alpha = 3}$, solved on the body of an elongated cell. **(b)** Final steady state of the 2D-FDM solution to the RD equations, initial condition $\boldsymbol{\alpha = 3}$, solved on a spherical mesh using finite differences.

Supplementary References

1. Boyce, W.E. & DiPrima, R.C. Elementary differential equations and boundary value problems, Edn. 6th ed. (J. Wiley, 1996).
2. Stroud, K.A. Fourier series and harmonic analysis. (Thornes, 1984).
